# Long-Lasting Gene Conversion Shapes the Convergent Evolution of the Critical Methanogenesis Genes

**DOI:** 10.1534/g3.115.020180

**Published:** 2015-09-16

**Authors:** Sishuo Wang, Youhua Chen, Qinhong Cao, Huiqiang Lou

**Affiliations:** * State Key Laboratory of Agro-Biotechnology and Ministry of Agriculture Key Laboratory of Soil Microbiology, College of Biological Sciences, China Agricultural University, Beijing 100193, China; † Department of Botany, University of British Columbia, Vancouver, British Columbia, V6T 1Z4, Canada; ‡ Department of Zoology, University of British Columbia, Vancouver, British Columbia, V6T 1Z4, Canada; § Department of Renewable Resources, University of Alberta, Edmonton, T6G 2H1, Canada

**Keywords:** gene conversion, concerted evolution, gene fusion, methyltransferase, methanogenesis

## Abstract

Methanogenesis and its key small-molecule methyltransferase Mtr complex are poorly understood despite their pivotal role in Earth’s global carbon cycle. Mtr complex is encoded by a conserved *mtrEDCBAFGH* operon in most methanogens. Here we report that two discrete lineages, *Methanococcales* and *Methanomicrobiales*, have a noncanonical *mtr* operon carrying two copies of *mtrA* resulting from an ancient duplication. Compared to *mtrA-1*, *mtrA-2* acquires a distinct transmembrane domain through domain shuffling and gene fusion. However, the nontransmembrane domains (MtrA domain) of *mtrA-1* and *mtrA-2* are homogenized by gene conversion events lasting throughout the long history of these extant methanogens (over 2410 million years). Furthermore, we identified a possible recruitment of ancient nonmethanogenic methyltransferase genes to establish the methanogenesis pathway. These results not only provide novel evolutionary insight into the methanogenesis pathway and methyltransferase superfamily but also suggest an unanticipated long-lasting effect of gene conversion on gene evolution in a convergent pattern.

Mtr is the key methyltransferase in the biological methanogenesis process, which plays a crucial role in Earth’s global carbon cycle and has a significant contribution to global warming (Gottschalk and Thauer 2001; Houghton *et al.* 2001). However, our knowledge of methanogenesis and its key small molecular methyltransferase remains very limited.

Methanogenesis is an ancient process performed exclusively by a particular group of anaerobic archaea called methanogens (Weiss and Thauer 1993; Bapteste *et al.* 2005; Gribaldo and Brochier-Armanet 2006; Liu and Whitman 2008). These methanogens are capable of catalyzing the formation of methane from low-molecular organic compounds like carbon dioxide and acetic acid, which is usually the final step in the decay of organic matter (Lelieveld *et al.* 1998; Houghton and Intergovernmental Panel on Climate Change Working Group I 2001). Methane (CH_4_) is not only the major component of clean natural gas but also a potent greenhouse gas. On the Earth, approximately 10^9-10^ tons of methane are biologically produced through methanogenesis each year, whereas 1–10% is estimated to be released into the atmosphere (Thauer 1998; Welander and Metcalf 2005).

Mtr, *N*^5^-methyltetrahydromethanopterin (CH_3_-H_4_MPT): Coenzyme M (CoM), catalyzes the second-to-last step of methanogenesis C1 pathway (Gottschalk and Thauer 2001). Mtr complex is composed of eight subunits (named as MtrA-H) with no homology between them (Harms *et al.* 1995). Genes encoding the eight subunits are located in the *mtrEDCBAFGH* operon (Harms *et al.* 1995). Among the eight subunits, MtrH is thought to be responsible for the energy-consuming transfer of the methyl group from methyl-tetrahydromethanopterin (H4MPT; in some species *N*5-methyltetrahydrosarcinapterin is the methyl donor) (Thauer 1998) to the corrinoid prosthetic group harbored by MtrA (Hippler and Thauer 1999), a protein anchored to cell membrane (Harms and Thauer 1996). The conformational change induced in MtrA via methylation and demethylation in turn enables the sodium pumper (probably MtrE) to pump Na^+^ extracellularly and helps establish the sodium ion gradient utilized in the ATP synthesis (Becher *et al.* 1992; Gottschalk and Thauer 2001; Lane and Martin 2012).

In this study, through a comprehensive phylogenetic and comparative genomic study of Mtr methyltransferase complex, we revealed an unexpected recurrent concerted evolution of *mtrA* genes, likely lasting throughout the whole history of the extant methanogens (over 2410 million years), after the convergent evolution of gene structure in three different archaeal lineages. Strikingly, the transmembrane domain of the same gene undoes significant divergent evolution very likely through domain shuffling and gene fusion. Additionally, we found an ancient nonmethanogenic origin of *mtrH* from a cobalamin-dependent methyltetrahydrofolate-homocysteine methyltransferase. Also, phylogenomic analyses detected homologs of *mtrA* and *mtrH* in nonmethanogens, which are probably involved in novel methyl-transfer pathways, implicating neo-functionalization after horizontal gene transfer (HGT) between methanogens and nonmethanogens. Our findings reveal the important roles of gene conversion and gene fusion in shaping the convergent evolution of methanogenesis genes and shed new insight into the evolution of the small-molecular methyltransferase gene superfamily.

## Materials and Methods

### Identification of homologous sequences

Protein sequences from Uniprot-Swiss annotated as subunits of Mtr were chosen as the seed sequences in BLASTP (Altschul *et al.* 1997) search. Homologs of *mtr* genes were identified with the E-value threshold of 1e-10 and BLOSUM62 matrix in all species with complete genome sequences available in RefSeq database (Pruitt *et al.* 2007). PSI-BLAST (Altschul *et al.* 1997) was used to search for remote homologs with default parameters in bacteria. The search was iterated until convergence. For homologs of Mtr proteins with a typical length less than 100 amino acids (MtrB, MtrF, MtrG), we used a different strategy to enhance the sensitivity of the search. We used seed sequences as the bait and searched for homologs in each order of methanogenic archaea. In this case, the hit with the lowest E value was then used to search for homologs in the same order and candidates with an E value lower than 1e-5 were considered as homologs. The taxonomic information of methanogenic archaea at order level was based on the work of Borrel *et al.* (2013), in which methanogens are classified into seven orders including six traditional orders and a recently proposed order referred to as Mx in this study for current communication (Borrel *et al.* 2013). The putative false-positive homologs were minimized with hmmscan implemented in HMMER3 (Eddy 1998) using the HMM profile of the corresponding domain. MeTr domain containing proteins shown in the phylogenetic tree were selected based on the list of representative species across different kingdoms of life (Richards *et al.* 2011) and clustered using Cd-hit (Li and Godzik 2006) to filter out highly redundant sequences. Sequence processing and format conversion were performed using custom Perl and Ruby (Goto *et al.* 2010) scripts.

### Phylogenetic reconstruction

Sequence alignment was performed using MUSCLE v3.8.31 (Edgar 2004) with default settings and edited manually with Bioedit (Hall 1999). Alignment was visualized with UGENE (Okonechnikov *et al.* 2012). Prior to phylogenetic reconstruction, jModelTest v2.1 (Darriba *et al.* 2012) and ProtTest v3.2 (Darriba *et al.* 2011) were used to find the most proper substitution model for DNA sequence alignment and protein sequence alignment using the Akaike Information Criterion (AIC), respectively.

Phylogenetic analyses were generated using maximum likelihood and Bayesian methods separately. The phylogenetic tree of each gene encoded by mtr operon was built based on protein sequences alignments using RAxML v7.3.9 (Stamatakis 2006) with 500 pseudo replicates of bootstrap sampling. To infer gene conversion events, maximum likelihood trees were reconstructed based on DNA sequences alignments with maximum likelihood and Bayesian methods, respectively. Maximum likelihood was reconstructed using Phyml 3.0 (Guindon and Gascuel 2003) with 1000 pseudo replicates of bootstrap sampling Bayesian phylogenetic analyses performed with Mrbayes v3.1 (Ronquist and Huelsenbeck 2003). MCMC chains were run for 10 million generations with sampling every 1000 generations. The average SD of split frequencies reached less than 0.002 when chains were summarized. The chains were thought to reach convergence. The first 25% of the sampled generations were discarded as burn-in and the rest were used in the calculation of posterior probabilities and consensus tree construction. Phylogenetic trees were visualized with MEGA v5 (Tamura *et al.* 2011), TreeGraph v2.0 (Stover and Muller 2010), and Figtree v3.1 (http://tree.bio.ed.ac.uk/software/figtree/). For DNA sequences utilized in gene conversion detection, maximum likelihood bootstrap support values and Bayesian inference posterior probability for each node were mapped using TreeGraph (Stover and Muller 2010) and checked manually. The phylogenetic history of all methanogens is based on the work of Borrel *et al.* (2013), in which all of the seven orders of methanogens are included. The divergence time of species was obtained from TimeTree (Battistuzzi and Hedges 2009; Kumar and Hedges 2011).

### Detection of gene conversion

To detect gene conversion events, phylogenetic trees were built using maximum likelihood and Bayesian methods based on DNA sequences separately as described above. Bootstrap values and Bayesian posterior probability were used to evaluate the confidence level of gene conversion events. Thus, if a gene is clustered with its paralogs from the same species instead of its orthologs from different species with a high bootstrap support and Bayesian posterior probability, it could be indicative of recent duplication or gene conversion at high frequency. For likelihood test of two competing topologies of the tree, we manually generated alternative tree topologies of *mtrA* for *Methanomicrobiales* and *Methanococcales* using Mesquite v2.75 (http://mesquiteproject.org). The *P*-values for different tests including Approximately Unbiased (AU) (Shimodaira 2002), Kishino-Hasegawa (KH) (Kishino and Hasegawa 1989), and Shimodaira-Hasegawa (SH) (Shimodaira and Hasegawa 1999) tests were calculated using CONSEL (Shimodaira and Hasegawa 2001) to indicate the likelihood of different tree topologies. Moreover, GENECONV (Sawyer 1989) was also applied to detect possible gene conversion events with default parameters except for 100,000 permutations that were performed to calculate the confidence for each gene conversion event. To get results with significant statistical support, only results with *P*-value smaller than 0.05 were considered to be positive gene conversion events. Furthermore, several analyzing methods implemented in RDP4 package (Martin *et al.* 2010) were used to verify the results of gene conversion, including RDP, BOOTSCAN, CHIMAERA, MAXCHI, SISCAN, and 3SEQ. The *P*-value for the null hypothesis of no recombination events is shown for each algorithm.

### Sequence similarity and Ka/Ks analysis

Pairwise sequence similarity was calculated with Water program implemented in the EMBOSS bioinformatic suite with default parameters (Rice *et al.* 2000). To perform calculate KA (nonsynonymous substitution rate), the nucleotide-coding sequences were aligned to the protein alignments using PAL2NAL (Suyama *et al.* 2006). Ka was estimated using CODEML from the PAML package v4.7 (Yang 2007).

### Structural analysis

FFAS (Jaroszewski *et al.* 2011), HHpred (Soding *et al.* 2005), and Phyre2 (Kelley and Sternberg 2009) were used in the fold recognition analysis to detect a proper model for homology modeling. Regions for 3D structure were chosen according to fold recognition result. The predicted model was built up by using Phyre2 based on the best reference. The refinement of the structure was done with ModRefiner (Xu and Zhang 2011). The quality of the predicted model was evaluated by two widely used programs PROQ (Wallner and Elofsson 2003) and ANOLEA (Melo *et al.* 1997). Mapping of consensus sequence to the surface of the structure was performed using Chimera (Pettersen *et al.* 2004). The superposition of the structure of MtrH and MeTr was performed using SuperPose (Maiti *et al.* 2004).

### Data availability

Supporting Information contains detailed descriptions of all supplemental files and Table S1, Table S2, Table S4, Table S5, Figure S1, Figure S2, Figure S3, Figure S4, Figure S5, Figure S6, Figure S7, Figure S8, Figure S9, along with supplemental references. Genomic context analysis of mtrH homologs are listed in Table S3. Parameters of natural habitat of all methanogenic archaea with complete genome sequences are summarized in Table S6.

## Results

### *Methanomicrobiales* and *Methanococcales* carry two *mtrA* paralogous genes with different structure in their *mtr* operons

**Figure 1:**
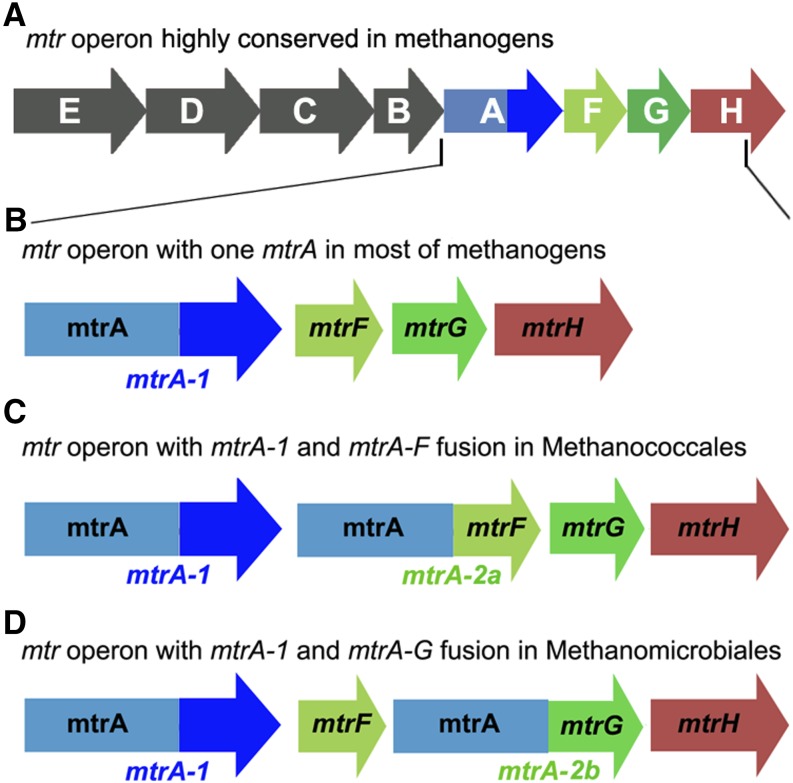
Schematic view of genomic context and gene structure of group I *mtrA*. (A) *mtrEDCBAFGH* operon in methanogens. (B) Methanogens usually encode one copy of a normal type of *mtrA* (*mtrA-1*) composed of an N-terminal MtrA domain labeled in teal and a C-terminal transmembrane domain in navy. (C and D) Besides *mtrA-1*, two orders of methanogens bear a second copy of *mtrA* designated as *mtrA-2* within the *mtr* operon. *mtrA-2* is the fusion type containing a MtrA domain fused at its C-terminus with a different transmembrane subunit of Mtr complex (*mtrA-F* fusion in *Methanococcales* and *Methanocella arvoryzae*; *mtrA-G* fusion in *Methanomicrobiales*). *mtrEDCB* in the upstream of *mtrA* are omitted for simplicity.

Mtr methyltransferase complex, as the key enzyme in methanogenesis, is encoded by a conserved *mtrEDCBAFGH* operon containing a single copy of each gene organized in the certain order in all methanogens (Figure 1, A and B) (Gottschalk and Thauer 2001), except for the recently proposed seventh order Mx (Paul *et al.* 2012; Iino *et al.* 2013). We identified two unconventional types of *mtr* operon carrying two copies of *mtrA* in *Methanomicrobiales* and *Methanococcales* orders by surveying all complete genomes in the NCBI RefSeq database (Figure 1, C and D). One copy is the normal type, *mtrA-1*, which presents widely throughout methanogens. It is composed of an N-terminal MtrA domain and a C-terminal transmembrane domain that may be required for anchoring MtrA protein to the cell membrane (Figure 1B) (Harms and Thauer 1997). Interestingly, the second copy of *mtrA* is not just a product of a simple gene duplication event. *mtrA-2* paralogous gene has a structure different from *mtrA-1*. It loses its C-terminal transmembrane domain and is located just downstream of *mtrA-1*. Moreover, *mtrA-2* became fused with downstream transmembrane genes *mtrF* and *mtrG* in *Methanococcales* and *Methanomicrobiales*, which were designated as *mtrA-2a* (Figure 1C) and *mtrA-2b* (Figure 1D), respectively. These results indicate that *mtrA-2* acquires a transmembrane domain distinct from *mtrA-1* through putative domain shuffling and gene fusion events.

To test the possible origin of the second *mtrA* copy, we constructed a phylogenetic tree of all seven methanogenic orders. *mtrA-2* only distributes in the two distantly related orders *Methanomicrobiales* and *Methanococcales* (Supporting Information, Figure S1), but not in the other five orders of methanogens. Such a discrete limited distribution does not favor the possibility that *mtrA-2* already exists in the last common ancestor of all methanogens, wherein at least five independent gene loss events should have occurred (Scenario I, Figure S1A). Furthermore, *mtrA-2a* and *mtrA-2b* show slightly different characteristics in gene structure (*mtrA-F* *vs.* *mtrA-G* fusion) and location (between *mtrA-1* and *mtrG* *vs.* between *mtrF* and *mtrH*) in the operon. These facts strongly suggest a lineage-specific origin of *mtrA-2a* and *mtrA-2b* (Scenario II, Figure S1B). Moreover, all 10 *mtrA-2a* orthologs were located in the syntenic genomic context with the same gene structure in the *mtr* operon of *Methanococcales*, and so were eight *mtrA-2b* orthologs in *Methanomicrobiales*. The highly conserved operon and gene structure strongly argue that the second copy of *mtrA* might be created in the common ancestor of each genus rather than in each species via recent gene duplication, fusion, and domain shuffling events. Therefore, it is more likely that *mtrA-2a* and *mtrA-2b* genes resulted from two independent duplications of *mtrA-1* followed by gene fusion and/or domain shuffling events that occurred in the common ancestors of *Methanomicrobiales* and *Methanococcales* lineages, respectively (Scenario II, Figure S1B).

### Long-lasting convergent concerted evolution between *mtrA-1/ mtrA-2* paralogs

**Figure 2:**
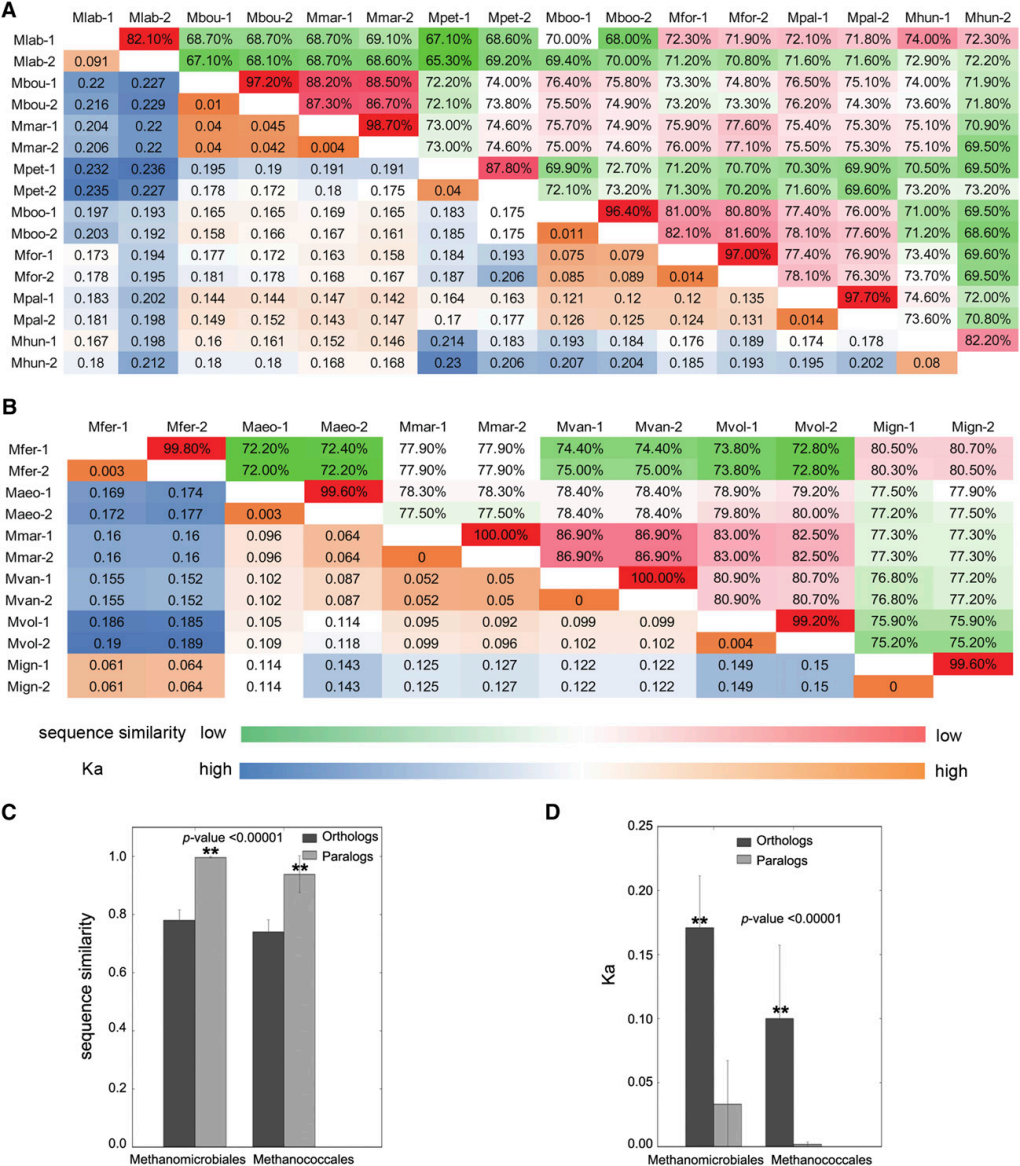
Heat map of sequence conservation between orthologs and paralogs of *mtrA* gene in *Methanomicrobiales* and *Methanococcales*. The pairwise sequence similarity (upper left) and Ka (lower right) ratio of two *mtrA* paralogs were calculated in *Methanomicrobiales* (A) and *Methanococcales* (B). Sequence similarity is indicated in the upper left side and is colored based on the nucleotide sequence identity. Red indicates a relatively high sequence identity and green stands for lower sequence identity. Ka ratios are shown in the lower left side and are colored based on the value of Ks from blue (higher Ka) to orange (lower Ka). Average DNA sequence similarity (C) and Ka (nonsynonymous substitution rate) (D) of orthologs and paralogs in *Methanomicrobiales* and *Methanococcales*. Abbreviations: Mfer, *Methanocaldococcus fervens* AG86; Mvul, *Methanocaldococcus vulcanius* M7; Maeo, *Methanococcus aeolicus* Nankai-3; Mmar, *Methanococcus maripaludis* S2; Mvan, *Methanococcus vannielii* SB; Mvol, *Methanococcus voltae* A3; Moki, *Methanothermococcus okinawensis* IH1; Mign, *Methanotorris igneus* Kol5; Mlab, *Methanocorpusculum labreanum* Z; Mbou, *Methanoculleus bourgensis* MS2; Mmar, *Methanoculleus marisnigri* JR1; Mpet, *Methanoplanus petrolearius* DSM 11571; Mboo, *Methanoregula boonei* 6A8; Mfor, *Methanoregula formicicum* SMSP; Mpal, *Methanosphaerula palustris* E1-9c; Mhun, *Methanospirillum hungatei* JF-1.

To examine the relationship between *mtrA-1* and *mtrA-2* outparalogs, we first analyzed the sequence similarity. Surprisingly, although *mtrA-1* and *mtrA-2* paralogs in the same species have distinct transmembrane domains, they bear a highly conserved MtrA domain at both amino acid (Figure S2) and nucleotide levels (Figure S3 and Figure S4). Next, we calculated the pairwise sequence similarity and substitution rate of MtrA domain of *mtrA* homologs using EMBOSS bioinformatic suite (Figure 2). Unexpectedly, the average sequence identities between *mtrA-1* and *mtrA-2* paralogs from the same species are 92.4% and 99.7% for *Methanomicrobiales* and *Methanococcales*, respectively, which are significantly higher than the values between their orthologs from other species (73.7% and 78.2%) (*P*-value < 0.00001) (Figure 2C). Meanwhile, the MtrA domain of *mtrA-1/2* paralogs shows significantly lower nonsynonymous substitution rates (0.033 and 0.002) than their orthologs (0.171 and 0.100) in *Methanomicrobiales* and *Methanococcales*, respectively (*P*-value < 0.00001) (Figure 2D). These results indicate that *mtrA-1* shares a much more similar MtrA domain with *mtrA-2* from the same species than orthologs from other species.

**Figure 3:**
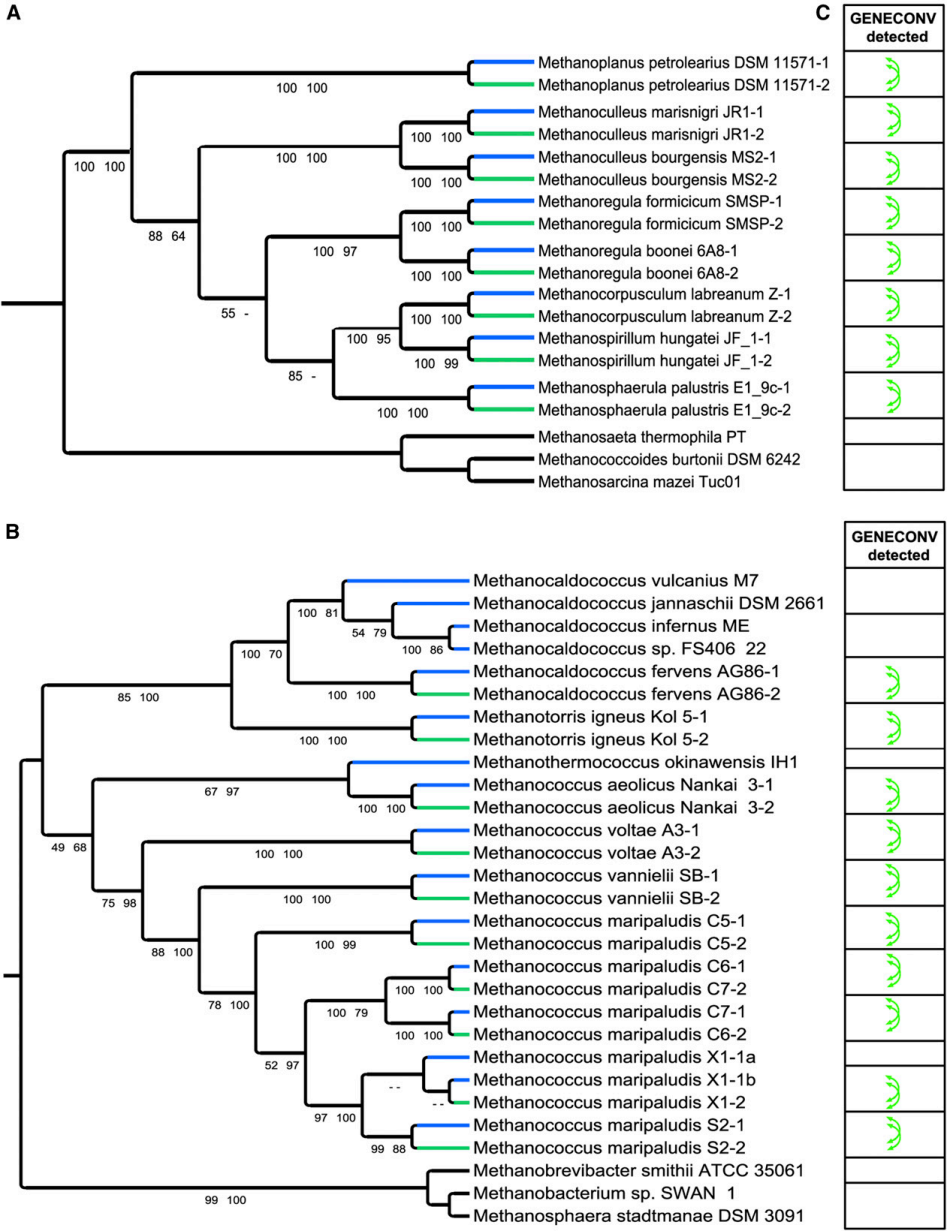
Unusual phylogenetic topology of two *mtrA* paralogs in *Methanomicrobiales* and *Methanococcales* orders. The cladograms depict the evolutionary relationship of *mtrA* homologs in *Methanomicrobiales* (A) and *Methanococcales* (B). For simplicity, the normal (*mtrA-1*) and fusion (*mtrA-2*) types of *mtrA* were labeled as -1 and -2 following the name of species, respectively. *mtrA-1* is presented in blue and *mtrA-2* is in green. The phylogenetic reconstruction was performed using DNA sequences of MtrA domain. The maximum likelihood (ML) bootstrap value was obtained with 1000 replicates and the Bayesian inference (BI) posterior probability was obtained with 10,000,000 MCMC generations run. Note that there are three copies of group I *mtrA* in *Methanococcus maripaludis X1*. Two normal types of *mtrA* gene were labeled as *mtrA-1a* and *mtrA-1b*. The support values are shown on the branch prior to each node (ML/BI). The bootstrap value and posterior probability lower than 50 are not shown in the tree.

To further test the close relationship between *mtrA-1* and *mtrA-2* paralogs, we reconstructed the phylogenetic trees of *mtrA* in *Methanococcales* and *Methanomicrobiales* using maximum likelihood (ML) and Bayesian posterior probability methods. Both trees showed that the two paralogs of *mtrA* existing in the same species in *Methanomicrobiales* and *Methanococcales* are clustered together with bootstrap values higher than 98 (Figure 3). To confirm the noncanonical topology of *mtrA* trees (Figure 3 and Figure S5A), we manually created an alternative tree topology where all *mtrA-1* orthologs group together while *mtrA-2* orthologs form a distinct clade (Figure S5B). Next, we compared the likelihood of two opposite topologies. The alternative scenario was statistically rejected with extremely low *P*-values for both *Methanomicrobiales* and *Methanococcales* by all topology comparison tests implemented in CONSEL (Figure S5D). These data support the unusual *mtrA* topology shown in Figure 3. Together, both sequence similarity and phylogenetic topology demonstrate that *mtrA-1* and *mtrA-2* paralogs within one species are more closely related to each other than to their orthologs in other species, indicating an unusual convergent evolutionary pattern of *mtrA-1* and *mtrA-2* paralogous genes.

**Figure 4:**
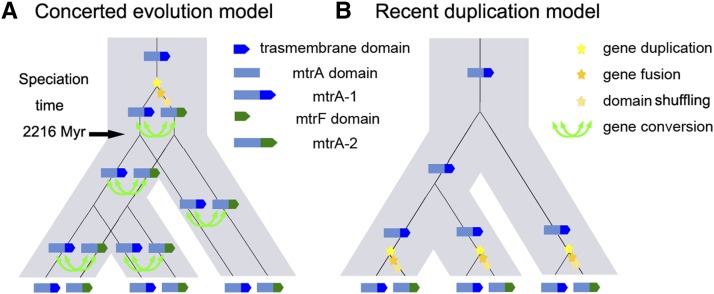
Two different evolutionary models regarding the observed phylogeny of *mtrA*. For clarity, only three species are shown. (A) *mtrA-2* is generated via an ancient gene duplication and domain shuffling event in the ancestor followed by frequent gene conversion (indicated as green arrow) in different lineages. (B) *mtrA-2* is generated via gene duplication and domain shuffling in different lineages multiple times. The star in yellow denotes the formation of *mtrA-2* through the duplication of *mtrA-1*. The stars in orange and light yellow denote the fusion between *mtrA-2* and *mtr-F/G* and the loss of the C-terminal transmembrane domain, respectively. Each branch in gray represents a speciation event.

The convergent evolution pattern of *mtrA-1/2* paralogs could be caused by two evolutionary mechanisms, concerted evolution (Figure 4A) or recent gene duplications (Figure 4B). The latter mechanism is much less likely because of the highly conserved syntenic genomic context and same gene structure of *mtrA-2a* and *mtrA-2b* in each lineage. *mtrA-2* was created via *mtrA-1* duplication, gene fusion, and domain shuffling events preceding the speciation of *Methanomicrobiales* and *Methanococcales* genera (Figure S1B, Scenario II). Therefore, the higher sequence similarity and phylogenies of *mtrA-1* and *mtrA-2* point to concerted evolution accomplished by gene conversion instead of recent duplication in each species (Scenario A, Figure 4A).

To confirm gene conversion events between *mtrA-1* and *mtrA-2*, we performed GENECONV analysis and obtained the statistical support for each event by 100,000 permutations (Mansai and Innan 2010). Only those with a *P*-value lower than 0.05 were considered to be positive conversion events. Gene conversion events were detected for all pairs of *mtrA-1/2* paralogs within the same species as identified in the phylogenetic trees (Figure 3C, Table S1). Moreover, these gene conversion events were further validated by several other computational tools implemented in RDP package as well (Table S2). These data indicate that the high sequence similarity and close relationship between *mtrA-1* and *mtrA-2* paralogs are maintained by recurrent gene conversion events, favoring the concerted evolution model (Scenario A, Figure 4A).

### Two novel subfamilies of *mtrA* gene with potential distinct functions

**Figure 5:**
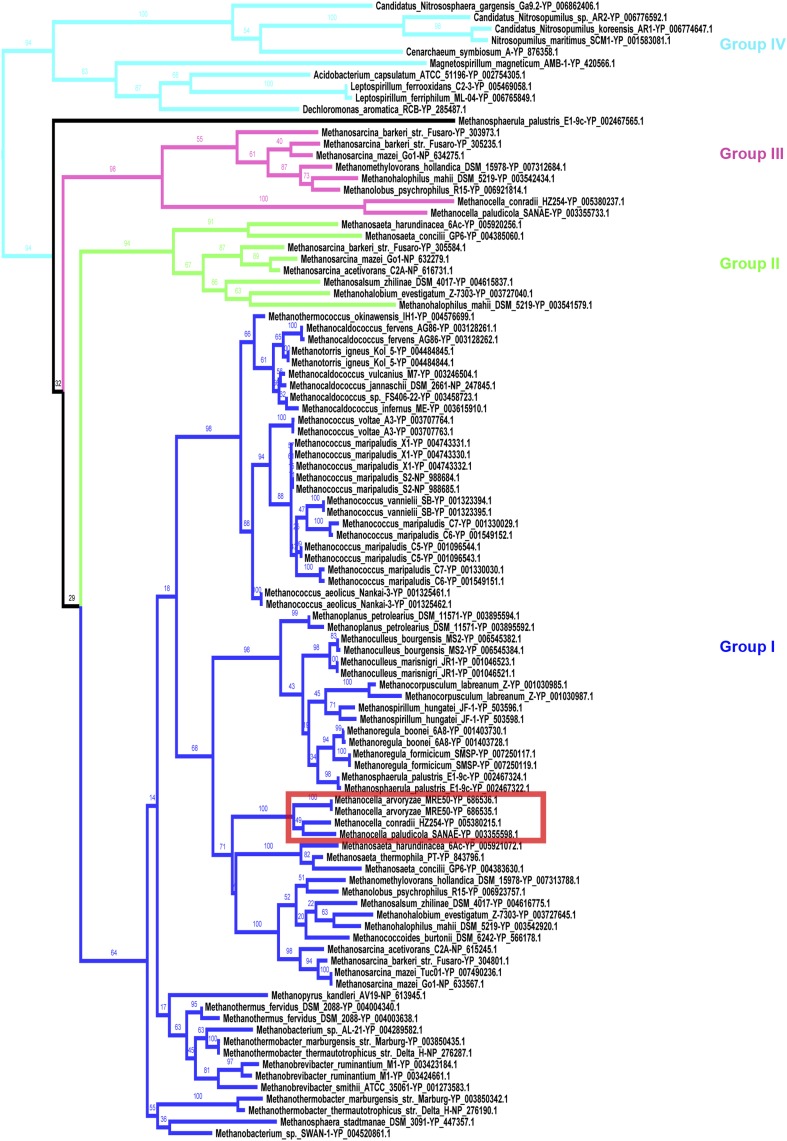
A more comprehensive phylogenetic analysis reveals new groups of *mtrA* gene. The maximum likelihood tree of *mtrA* was constructed with RAxML. The substitution model was determined by ProtTest prior to the phylogenetic reconstruction. Homologs of *mtrA* were classified into four groups (subfamilies). Group I, group II, group III, and group IV of *mtrA* are colored blue, green, pink, and light blue, respectively. Group I is the dominant type found in most methanogens with a C-terminal transmembrane domain. Homologs in groups II and III are only found in *Methanosarcinales* and/or Methanocellales. Group IV *mtrA* is only distributed in nonmethanogens. The number on the scale bar indicates the number of substitutions per amino acid site. The number next to each node indicates the bootstrap value. *Methanocella arvoryzae*, which has two group I *mtrA* paralogs, was marked in red square.

To examine the evolutionary scenario of *mtrA-1* and *mtrA-2* in a broader scope, phylogenetic analysis was expanded to all homologs of *mtrA* identified in all complete genomes. The tree obtained by comprehensive phylogenetic analysis clearly revealed four groups of *mtrA* (Figure 5). Among them, two new groups, as defined as group III and group IV, have not been identified previously.

The biggest clade composed by the majority of *mtrA* genes was defined as group I. All group I *mtrA* genes are located within the *mtr* operon, which exists in all methanogens except the Mx order (Borrel *et al.* 2013). *Methanomicrobiales* and *Methanococcales* species carried two copies of group I *mtrA*, *mtrA-1*, and *mtrA-2* as mentioned above. Again, these two paralogs clustered together, which recapitulates the noncanonical topology in Figure 2.

Interestingly, the other three groups of *mtrA* lost the C-terminal transmembrane domain in group I counterparts. Furthermore, according to the phylogenetic tree, all these *mtrA* genes appeared with long branches apart from group I homologs. Group II *mtrA* was only distributed in Methanosarcinales. In addition, it forms an operon together with a conserved methanogenesis gene *mtxX* and a *mtrH* homolog *mtxH*. It has been proposed that together with *mtxX*, group II *mtrA* and/or *mtxH* might participate in the methyltransfer from trimethylamine or methanol to tetrahydromethanopterin, a methanogenesis pathway unique to Methanosarcinales (Harms and Thauer 1997).Group III *mtrA* exists in Methanosarcinales and Methanocellales lineages and lacks apparent conserved genomic context. We are able to find some *mtrA* homologs sporadically distributed in nonmethanogenic bacteria and archaea, composing group IV *mtrA* (Figure 5). The patchy distribution pattern indicates that these organisms may obtain *mtrA* homologs by horizontal gene transfer (HGT). These data show that groups II, III, and IV *mtrA* distinguish themselves from group I homologs in many aspects, including sequence similarity, gene structure, and genomic context. This suggests that in contrast to the concerted evolution of *mtrA-1* and *mtrA-2* in *Methanomicrobiales* and *Methanococcales*, other groups of *mtrA* undergo sequence and potential functional diversification.

### Another putative convergent concerted evolution example in *Methanocella arvoryzae*

In the comprehensive phylogenetic tree described above, we noticed that *Methanocella arvoryzae* is the only organism outside the *Methanomicrobiales* and *Methanococcales* orders encoding two copies of group I *mtrA* (Figure 5, red square). Like *Methanococcales*, *Methanocella arvoryzae* has a *mtrA-F* fusion type (*mtrA-2a*) downstream of *mtrA-1* in the *mtr* operon. More interestingly, the two *mtrA* paralogs in *Methanocella arvoryzae* showed high sequence similarity and were clustered into the same clade in the ML tree, resembling the convergent pattern as *mtrA-1* and *mtrA-2* paralogs in *Methanococcales* and *Methanomicrobiales*.

One possibility is that it is due to a recent duplication of *mtrA* followed by the loss of C-terminal transmembrane domain and fusion with *mtrF* (Figure S6A). However, it is worth noticing that group III *mtrA* is missing in this organism while it exists in all other species from the same order. This raised another possibility that group III *mtrA* might be fused with *mtrF* after a translocation event and got converted by *mtrA-1* later (Figure S6B). This scenario couples the loss of group III *mtrA* and the high similarity between two *mtrA* paralogs and avoids assuming the duplication of *mtrA-1*, the loss of the transmembrane domain of *mtrA-1*, and the loss of group III *mtrA*. Thus, this scenario is more parsimonious and likely represents the third example of independent origin of the chimeric *mtrA-F* gene through concerted evolution.

### An ancient origin of MtrH from the MeTr domain of B12-dependent methionine synthase (MetH)

In Mtr complex, MtrH is the methyltransferase responsible for the transfer of methyl group between CH_3_-H_4_MPT and corrinoid prosthetic group harbored by MtrA (Hippler and Thauer 1999). The prevalence and crucial role of MtrH among methanogens aroused our interest to pursue a clearer evolutionary map of *mtrH* in a context of whole methyltransferase superfamily. Surprisingly, a PSI-BLAST search against the NCBI RefSeq database hit numerous methyltransferases with sequence identity of 30% with *mtrH* in the second round of iteration. The PSI-BLAST was not stopped until no more hits were detected at the e-value cutoff of 1e-3. Vitamin B12-dependent methionine synthase (MetH) and 5-methyltetrahydrofolate–homocysteine methyltransferase appeared to be mostly enriched among all of the hits. The amino acid sequence alignment of MtrH and MeTr revealed a conserved region limited to the pterin ligand binding MeTr domain of MetH and 5-methyltetrahydrofolate–homocysteine methyltransferase (Figure S7). To further illustrate the relationship between MtrH and MeTr from a structural perspective, fold recognition search was performed using MtrH from *Methanococcus jannaschii*, the first sequenced archaeon. In agreement with amino acid sequence similarity, all three fold recognition analyses (FFAS, HHpred, and Phyre2) consistently reported a region with similarity to the MeTr domain in B12-dependent methionine synthase (MetH) and dihydropteroate synthetase (DHPS), thus pointing to a remote homologous relationship between MtrH and MeTr domain.

**Figure 6:**
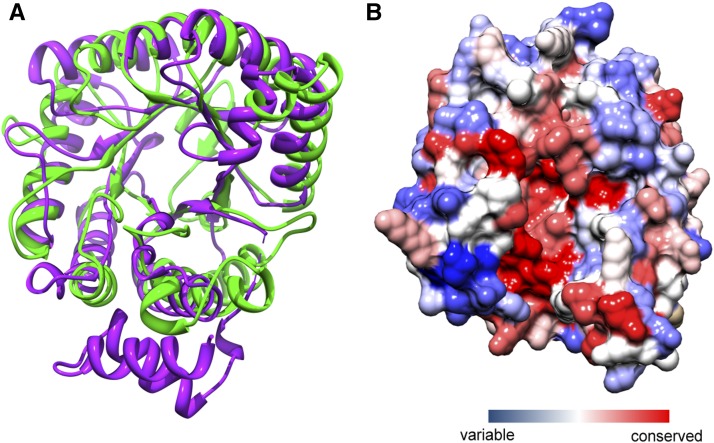
Predicted 3D structure of MtrH of *Methanococcus jannaschii*. (A) The superimposition of MtrH (green) and MeTr (purple). The amino acids at the position of 50-271 of the best-aligned protein (PDB ID: 3K13) are chosen in structural comparison. The structural model of MtrH shows a TIM-barrel fold. (B) The structure of MtrH is colored based on the evolutionary conservation information. The red represents the most conserved residues and the blue indicates the least conserved residues.

In addition, we built a homology modeling of the MtrH via Phyre2. The N-terminal region (a.a.11-299) was used in 3D modeling because it shows relatively high similarity in both PSI-BLAST and folding recognition search. The predicted MtrH tertiary structure was supported by both computational programs PROQ (Wallner and Elofsson 2003) and ANOLEA (Melo *et al.* 1997). MtrH presented a TIM-barrel structure, which closely resembles the structural feather of MeTr domain (Figure 6A). A more detailed comparison between the MtrH and MeTr domain at the 3D level revealed a highly conserved region (colored red in Figure 6B) corresponding to the ligand binding motifs of MeTr domain, indicating the potential conserved functions on ligand binding between MtrH and MeTr.

Furthermore, we constructed an ML tree of MtrH/MeTr domain-containing proteins. The whole MtrH group was clustered together with groups of MetH and 5-methyltetrahydrofolate–homocysteine methyltransferase (Figure S8). Both MetH and 5-methyltetrahydrofolate–homocysteine methyltransferase are present in numerous organisms from different kingdoms, implying a common ancient origin of MtrH and MeTr domain-containing proteins.

Taken together, these data suggest an ancient nonmethanogenic origin of *mtrH*, which means that MeTr domain of B12-dependent methionine synthase and 5-methyltetrahydrofolate–homocysteine methyltransferase might evolve to become MtrH and be utilized by methanogens for methanogenesis.

### Massive bidirectional interdomain HGTs of *mtrH*

**Figure 7:**
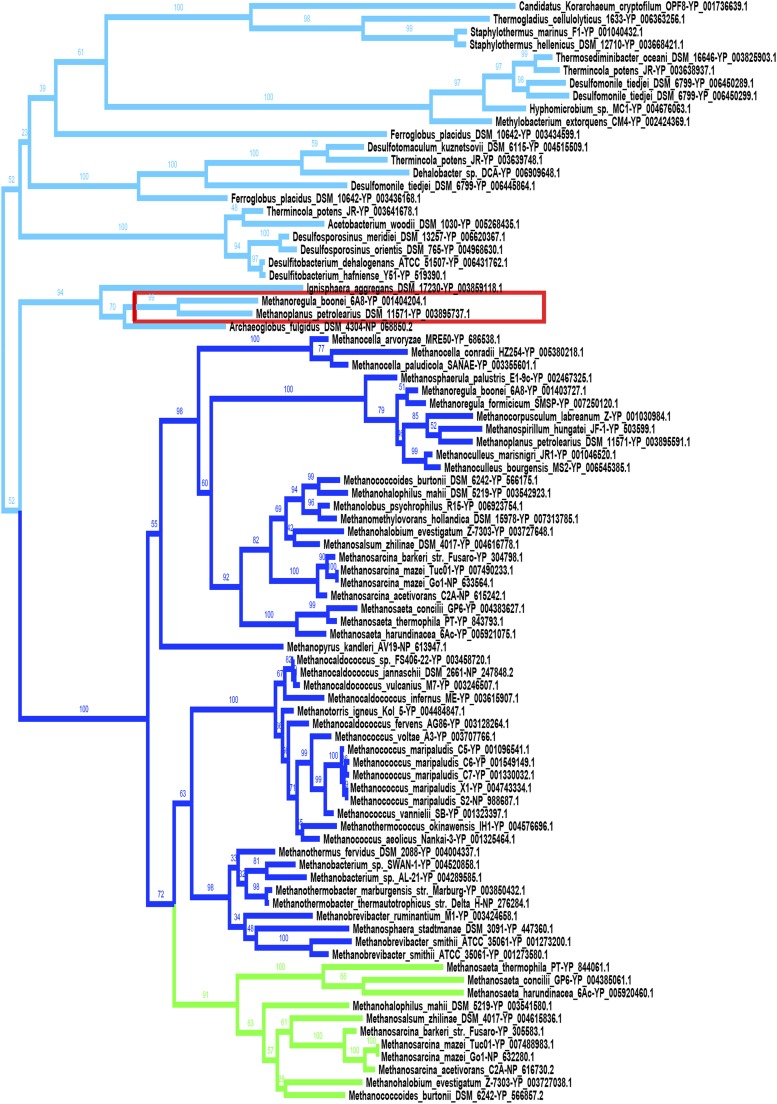
Massive horizontal gene transfer between methanogens and nonmethanogens in the evolution of *mtrH*. The maximum likelihood tree of *mtrH* was constructed with RAxML. ProtTest was utilized to estimate the substitution model used in the phylogenetic reconstruction. *mtrH* homologs correspond to three subgroups. Subgroups I, II, and III *mtrH* homologs are colored deep blue, green, and light blue, respectively. The majority is subgroup I, which is located in *mtrEDCBAFGH* operon. Subgroup II *mtrH* is located in *mtxXAH* operon, with group II *mtrA* only distributed in *Methanosarcinales*. Subgroup III contains all *mtrH* homologs from nonmethanogens, and two homologs from methanogens also belong to this group (in the red square).

If we took a closer view of MtrH branch, it comprises three subgroups (Figure 7). Subgroup I *mtrH* is located in *mtr* operon involved in C1 methanogenesis pathway. Subgroup II forms an operon with group II *mtrA* and might participate in other methanogenesis pathways (Harms and Thauer 1997). *mtrH* homologs are also found in several distantly related nonmethanogens, which are named subgroup III. They form a separate clade in the phylogenetic tree (Figure 7). The topology of the tree and patchy distribution of subgroup III *mtrH* among very distantly related organisms tends to suggest that some nonmethanogens might acquire *mtrH* homologs from methanogenic archaea and that massive HGT events occurred among nonmethanogens.

Interestingly, *mtrH* in nonmethanogens show a conserved genomic context with other methyltransfer-related genes (Table S3). The operons include homologs of *mta*, which are thought to catalyze methanogenesis from methanol. This implicates functional constraints on the structure of operons containing *mtrH* homologs in nonmethanogens. It would be postulated that *mtrH* are likely neo-functionalized and involved in novel methyltransfer processes after HGT in many nonmethanogenic lineages. More interestingly, several prokaryotic species that carry *mtrH* homologs are able to utilize chloromethane as carbon and energy source (highlighted in Table S3). Considering the functional links between *mtrH* and its neighbor genes in bacteria (Gao and Gupta 2007), it is likely that these bacteria acquired *mtrH* homologs via HGT to help reduce methyl halides.

Finally, it is worth noting that two *mtrH* homologs from methanogens, *Methanoplanus petrolearius DSM 11571* and *Methanoregula boonei 6A8*, group with *mtrH* homologs from nonmethanogens supported by bootstrap values of 94 and 79 (Figure 7). Additionally, the genomic context is conserved among these species as well (Table S3). This may represent a rare case of reverse horizontal transfer of *mtrH* homologs from nonmethanogens to methanogens. Taken together, the phylogeny of *mtrH* suggests massive bidirectional gene transfer between methanogens and nonmethanogens and within nonmethanogens.

## Discussion

Gene conversion, the process of nonreciprocal exchange between two DNA segments (Liao 1999), can cause the concerted evolution of different sequences, thereby leading to functional conservation or redundancy of duplicated genes (Nei and Rooney 2005; Innan and Kondrashov 2010). In this study, through comprehensive phylogenetic and rigorous computational analyses, we found an exceptional long-lasting recurrent gene conversion mechanism that constrains the divergence of *mtrA-1* and *mtrA-2* paralogs with different gene structure in three distinct methanogenic archaeal lineages in parallel. Such a unique evolutionary process may be important for *mtrA* gene function and may be driven by adaptation to survive in a methanogenesis manner under some particular environment.

### *mtrA-1*/*mtrA-2* paralogs represents a unique example of convergent concerted evolution

In bacteria, concerted evolution of paralogs should occur through homologous recombination, which is usually considered to have a similar outcome of eukaryote’s gene conversion. Interestingly, only the highly conserved MtrA domain in *mtrA-2* is overwritten by copying the counterpart sequence from *mtrA-1*. MtrA-2 obtained a transmembrane domain distinct from MtrA-1 through domain shuffling and gene fusion, thus leading to a divergent pattern with MtrA-1 paralog in transmembrane domain. As far as we know, the evolutionary scenario of MtrA-2 reported here represents the first example that convergent and divergent evolution occur in the two distinct domains of the same gene through distinct mechanisms, respectively.

Although *mtrA-1* and *mtrA-2* paralogs in the same species have distinct transmembrane domains, they bear a significantly more similar MtrA domain than their orthologs in different species at both levels of nucleic acid and amino acid sequence. These data point to a unique convergent evolution within a certain domain of two paralogous genes. However, the gene structure and genomic/operon context of *mtrA-2* are highly conserved at the genus level, indicating a convergent pattern of *mtrA-2* evolution in each lineage as well. Such a convergent pattern is ascribed to a concerted evolution (Figure 4A) rather than recent gene duplication (Figure 4B). It is worth mentioning that although a recent duplication model is not favored by the same gene structure and highly conserved genomic/operon context at the genus level, we cannot completely rule out this possibility. However, if the high similarity between *mtrA-1*/*2* paralogs were due to recent duplication, then first we would have to assume at least 10 and eight independent duplication gene fusion domain shuffling events in *Methanococcales* and *Methanomicrobiales*, respectively. Second, we would also have to assume such a complicated process should have occurred constantly in each species belonging to the same genus.

### Exceptional long-lasting gene conversion events underlie *mtrA-1*/*mtrA-2* concerted evolution

Gene conversion events should predate the speciation events in the orders *Methanomicrobiales* and *Methanococcales* because concerted pattern and gene conversion were detected in almost all organisms in these two orders. Moreover, given the fact that *mtrA-1* and *mtrA-2* share significantly high similarity in their MtrA domain at the nucleotide sequence level, it is very likely that concerted evolution is still an ongoing process in extant methanogens. According to the archaeal TimeTree (Battistuzzi and Hedges 2009; Kumar and Hedges 2011), gene conversion events between *mtrA-1* and *mtrA-2* can thus be estimated to last for more than 2410 Myr (million years) in *Methanomicrobiales* and 2216 Myr in *Methanococcales* species (Table S4, Figure S9). This case of gene conversion, to our knowledge, represents one of the most long-lasting concerted evolution events that have been reported so far (Table S4 and Table S5). It is more intriguing given that it happens independently in two distinct lineages and that both *mtrA* paralogs are essential to the cell growth and/or methanogenesis (Sarmiento *et al.* 2013). The MtrA domain is essential for most methanogens to thrive through an extremely ancient and highly conserved methanogenesis reaction. Therefore, we suggest that it is most likely that gene conversion may contribute to the removal of deleterious mutations and the spread of beneficial mutations (Mano and Innan 2008; Fawcett and Innan 2011). The recurrent evolution of gene conversion becomes a good strategy to maintain a highly conserved MtrA domain in two *mtrA* paralogs. This might be particularly important for archaea where recombination through sexual reproduction is absent (Breuert *et al.* 2006). Also, it is known that for genes involved in protein complexes like Mtr, the existence of divergent paralogs might introduce problems as other subunits may have to adapt to different copies of the same gene (Baker *et al.* 2013). The homogenization of paralogous sequences via gene conversion may thus help to avoid this problem in the Mtr complex with multiple protein–protein interactions.

However, the C-terminal transmembrane domain only exists in group I *mtrA* belonging to Mtr complex, but not other groups of *mtrA*. This suggests that the transmembrane domain should be indispensable to Mtr complex, and thus methanogenesis capability. One possibility is that the transmembrane domain might be involved in sensing different ligands or other environmental cues important for methanogenesis. Among species with habitat data currently available, most organisms where gene conversion of *mtrA* occurred were found to be mesophiles (Table S6). However, not all mesophilic methanogens carry *mtrA-2*, implying that there could be some unknown environmental factor(s) contributing to such extremely long-lasting concerted evolution and determining the distinct transmembrane domains of *mtrA-1/2*.

In summary, we suggest that the unusual evolutionary pattern of MtrA paralogs represents a novel example that gene conversion may help unsexual organisms escape from “Muller ratchet” (Muller 1964) by allowing for more efficient spread of beneficial mutations and removal of deleterious mutations after the convergent domain shuffling in different lineages.

### The nonmethanogenic origin of MtrH From MeTr

Another interesting finding is that the methyltransferase MtrH required for methanogenesis may be traced back to a nonmethanogenic origin. The homologous relationship between MtrH and MeTr observed in this study provides a case of the transfer of nonmethanogenic genes to methanogenesis genes and indicates the utilization of preexisting genes in the genome in the establishment of the methanogenesis pathway. This is in agreement with previous findings that two enzymes involved in the acetoclastic methanogenesis pathway may originate from *Firmicutes* (Fournier and Gogarten 2008). All these data provide evidence to support the recent hypothesis that the origin of some methanogenesis genes could be dated back to the last common ancestor of archaea (LCA) based on an extensive genomic analysis (Chistoserdova 2013).

## 

## Supplementary Material

Supporting Information
