# Supplementary material for: Long-Lasting Gene Conversion Shapes the Convergent Evolution of the Critical Methanogenesis Genes
Source: G3 (Bethesda). 2015 Sep 16;5(11):2475–86. doi: 10.1534/g3.115.020180 (PMC4632066; doi:10.1534/g3.115.020180)
Supplement: Supporting Information [file supp_5_11_2475__index.html]

Long-Lasting Gene Conversion Shapes the Convergent Evolution of the Critical Methanogenesis Genes — Supporting Information 

# Long-Lasting Gene Conversion Shapes the Convergent Evolution of the Critical Methanogenesis Genes

## Supporting Information for Wang *et al.*, 2015

**Files in this Data Supplement:**

- Supporting Information - Tables S1-S6, Figures S1-S9, and Supplementary References (PDF, 3 MB)
- Table S1 - (Related to Figures 3 & 4): Gene conversion events detected by GENECONV between *mtrA-1* and *mtrA-2* paralogous genes in different species. (PDF, 27 KB)
- Table S2 - (Related to Figures 3 & 4): Recombination tests by different algorithms implemented in the RDP4 package. (PDF, 59 KB)
- Table S4 - (Related to Figures 3 and 4): A summary of the duration of some known gene conversion events. (PDF, 21 KB)
- Table S5 - (Related to Figures 3 and 4): A summary of gene conversion events in genome-wide assays. (PDF, 15 KB)
- Figure S1 - (Related to Figure 1). Evolutionary scenarios of the convergent evolutionary pattern of *mtrA-2*. (PDF, 564 KB)
- Figure S2 - (Related to Figure 2). Amino acid sequence alignment of mtrA domainin (*A*) Methanomicrobiales and (*B*) Methanococcales. (PDF, 656 KB)
- Figure S3 - (Related to Figure 2): Nucleotide sequence alignment of mtrA domainin Methanomicrobiales. (PDF, 856 KB)
- Figure S4 - (Related to Figure 2): Nucleotide sequence alignment of mtrA domain in Methanococcales. (PDF, 900 KB)
- Figure S5 - (Related to Figure 3 and Table S1). (*A*) The expected topology if gene conversion happens throughout all lineages. (*B*) The expected topology where no gene conversion happens. (*C*) The tree of species. (*D*) Statistic tests of two different topologies. (PDF, 573 KB)
- Figure S6 - (Related to Figure 5). Two different scenarios in regards to the additional copy of group I *mtrA* in *Methanocella arvoryzae*. (PDF, 531 KB)
- Figure S7 - (Related to Figure 6). Multiple amino acid sequence alignment of MtrH and its remote homologs. (PDF, 778 KB)
- Figure S8 - (Related to Figure 7). A ML tree of MtrH and MeTr domain-containing proteins. (PDF, 925 KB)
- Figure S9 - (Related to Figure 4). An overview of the evolutionary scenario of *mtrA-1/2* in all methanogens. (PDF, 587 KB)
- Table S3 - (Related to Figure 7): Genomic context analysis of *mtrH* homologs. (.xlsx, 27 KB)
- Table S6 - (Related to Figures 2 & 3): Summary of known parameters of natural habitat of all methanogenic archaea with complete genome sequences. (.xlsx, 14 KB)
