# Supplementary material for: Long-Lasting Gene Conversion Shapes the Convergent Evolution of the Critical Methanogenesis Genes
Source: G3 (Bethesda). 2015 Sep 16;5(11):2475–86. doi: 10.1534/g3.115.020180 (PMC4632066; doi:10.1534/g3.115.020180)
Supplement: Supporting Information [file supp_g3.115.020180_020180SI.pdf]

G3· Genes | Genomes | Genetics

Long-lasting gene conversion shapes the convergent evolution of the critical methanogenesis genes

Sishuo Wang[1, 2, *], Youhua Chen[3, 4], Qinhong Cao[1], Huiqiang Lou[1, *]

1 State Key Laboratory of Agro-Biotechnology and Ministry of Agriculture Key Laboratory of Soil Microbiology, College of Biological Sciences, China Agricultural University, 2 Yuan-Ming-Yuan West Road, Beijing 100193, China

2 Department of Botany, University of British Columbia, Vancouver, BC, V6T 1Z4, Canada

3 Department of Zoology, University of British Columbia, Vancouver, BC, V6T 1Z4, Canada

4 Department of Renewable Resources, University of Alberta, Edmonton, T6G 2H1, Canada

* To whom correspondence should be addressed. Email: wangsishuo@yeah.net (S. Wang)

Email: lou@cau.edu.cn (H. Lou)

DOI: 10.1534/g3.115.020180

Table S1 (Related to Figure 3&4): Gene conversion events detected by GENECONV between *mtrA-1* and *mtrA-2* paralogous genes in different species. *P*-value obtained from 100,000 permutations is shown as indicator of the confidence level of each gene conversion event. Only those with *P*-values lower than 0.05 are considered as potential gene conversion events and summarized in the table. * SIM (Similarity), BC KA (Bonferroni-corrected Karlin-Altschul)

Organisms	SIM* *P*-value	BC KA *P*-value	Aligned begin	Aligned end	Length	No. of polymorphisms	Total differences	Mismatch penalty
Methanoculleus marisnigri JR1	0	0	1	503	503	268	103	None
Methanosphaerula palustris E1_9c	0	0	61	478	418	222	116	None
Methanoregula formicicum SMSP	0	0	77	434	358	187	114	None
Methanoculleus bourgensis MS2	0	0	1	356	356	182	112	None
Methanoregula boonei 6A8	0	0	275	524	250	154	119	None
Methanocaldococcus fervens AG86	0	0	1	532	532	268	113	None
Methanococcus voltae A3	0	0	1	490	490	242	109	None
Methanococcus vannielii SB	0	0	1	551	551	287	93	None
Methanococcus maripaludis C5	0	0	1	551	551	287	89	None

Methanococcus maripaludis S2	0	0	1	551	551	287	88	None
Methanococcus maripaludis X1	0	0	1	534	534	270	89	None
Methanococcus aeolicus Nankai_3	0	0	1	454	454	219	105	None
Methanotorris igneus Kol 5	0	0	1	416	416	194	108	None
Methanococcus maripaludis C7	0.0031	0.0105 1	288	362	75	35	157	None

Table S2 (Related to Figure 3&4): Recombination tests by different algorithms implemented in the RDP4 package. The *P*-value is shown if a recombination between two paralogs from the same species is detected for each algorithm. Otherwise indicated as 'NS' (not significant).

Species	RDP	Bootscan	Maxchi	Chimaera	SiSscan	3Seq
Methanoculleus marisnigri JR1	7.42E-23	7.74E-14	1.04E-18	7.24E-16	2.13E-19	8.76E-32
Methanosphaerula palustris E1-9C	3.98E-20	7.68E-24	2.80E-16	2.05E-16	1.55E-15	5.17E-31
Methanoregula formicicum SMSP	1.52E-18	1.01E-23	9.22E-19	2.39E-16	4.00E-19	4.34E-32
Methanoregula boonei 6A8	4.25E-15	5.40E-17	3.22E-17	7.90E-17	4.71E-11	7.17E-27
Methanoplanus petrolearius DSM	1.10E-09	3.84E-08	1.29E-09	1.71E-11	2.99E-08	6.22E-14
Methanocorpusculum labreanum Z	4.25E-08	2.17E-05	1.21E-12	5.06E-11	1.11E-06	3.10E-15
Methanospirillum hungatei JF-1	9.57E-06	0.000143	2.73E-08	3.56E-10	3.45E-10	8.46E-10
Methanocaldococcus fervens AG86	2.22E-22	4.26E-28	7.59E-19	1.51E-14	7.46E-21	2.88E-25
Methanotorris igneus Kol 5	5.27E-22	1.07E-18	5.72E-20	3.74E-14	3.94E-23	3.96E-26
Methanococcus maripaludis S2	3.89E-20	1.41E-16	2.83E-17	5.29E-17	1.29E-11	6.00E-29
Methanococcus vannielii SB	1.66E-17	3.43E-07	1.82E-12	1.61E-14	6.49E-18	2.72E-21
Methanococcus	2.99E-10	1.21E-12	5.86E-16	4.80E-13	9.45E-16	2.68E-15

voltae A3						
Methanococcus aeolicus Nankai-3	1.26E-09	4.06E-12	3.97E-05	3.01E-11	2.49E-25	2.47E-10

Table S3 (Related to Figure 7): Genomic context analysis of *mtrH* homologs. Four upstream and four downstream genes for each *mtrH* homolog were retrieved to search for adjacent genes involved in methyl-transfer processes. Potential methyl-transfer related genes outside of the operon *mtrEDCBAFGH* are highlighted. Organisms capable to use methyl halides are highlighted as well. (.xlsx)

Available for download at www.g3journal.org/lookup/suppl/doi:10.1534/g3.115.020180/-/DC1

Table S4 (Related to Figure 3 and 4): A summary of the duration of some known gene conversion events. The duration time of each gene conversion event was estimated based on TreeTime if colored in blue, or from the indicated literatures.

Genes	Domain	Duration of gene conversion (Myr)	Literatures
***mtrA-1/mtrA-2a* in Methanococcales**	**Archaea**	**> 2216**	**This study**
***mtrA-1/mtrA-2b* in Methanomicrobials**	**Archaea**	**> 2410**	**This study**
tuf	Bacteria	> 2500	(Lathe and Bork 2001; Kondrashov, Gurbich and Vlasov 2007)
gadA/B	Bacteria	< 100	(Bergholz, et al. 2007)
type IV secretion system genes	Bacteria	< 0.5	(Nystedt, et al. 2008)
engrailed-family genes in beetles	Eukarya	~ 360	(Peel, Telford and Akam 2006)
silk genes in spiders	Eukarya	> 240	(Garb, et al. 2007)
Csd in ant/bee	Eukarya	115	(Schmieder, Colinet and Poirie 2012; Privman, Wurm and Keller 2013)
spp120 in Cichlid fish	Eukarya	100	(Gerrard and Meyer 2007)
vitellogenin genes in mosquito	Eukarya	100	(Chen, et al. 2010)
Nspb and *Nspc* gene families in C. elegans	Eukarya	50	(Thomas 2006)
dumpy genes in Drosophila	Eukarya	30	(Carmon, et al. 2007)
xq28 in primates	Eukarya	25	(Bagnall, et al. 2005)

siglec-11/16 in Hominins	Eukarya	1-1.2	(Wang, et al. 2012)

Table S5 (Related to Figure 3 and 4): A summary of gene conversion events in genome-wide assays. The duration time of gene conversion in each organism was estimated based on TreeTime if colored in blue, or from the indicated literatures.

**Taxa**	**No. of gene pairs/gene families**	**gene conversion lasting time (Myr)**	**Literatures**
yeast	55	< 100	(Gao and Innan 2004)
primates	43	< 20-95	(Ezawa, et al. 2010)
rice	244	< 41.3	(Wang, et al. 2009)
sorghum	210	< 41.3	(Wang, et al. 2009)
rat and mouse	488	< 25.4	(Ezawa, OOta and Saitou 2006)

Table S6 (Related to Figure 2 &3): Summary of known parameters of natural habitat of all methanogenic archaea with complete genome sequences. The data were collected from NCBI RefSeq database if not particularly indicated. Organisms with detected gene conversion between two paralogs of *mtrA* are highlighted. (.xlsx)

Available for download at www.g3journal.org/lookup/suppl/doi:10.1534/g3.115.020180/-/DC1

Figure S1 (Related to Figure 1)

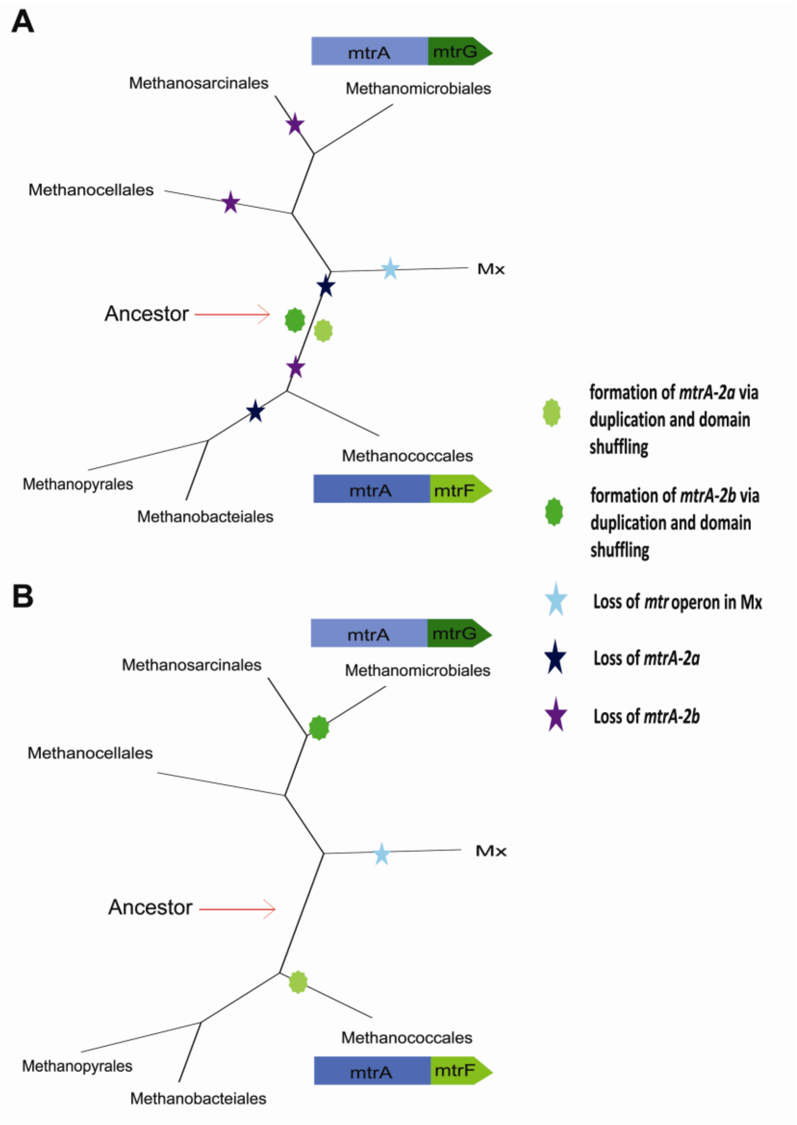

Figure S1 (Related to Figure 1). Evolutionary scenarios of the convergent evolutionary pattern of *mtrA-2*. (A) Scenario I: *mtrA-2* originated in Methanomicrobiales and Methanococcales independently. (B) Scenario II: *mtrA-2* originated in the common ancestor of all methanogens followed by several gene loss events in different lineages for at least five times.

Figure S2 (Related to Figure 2)

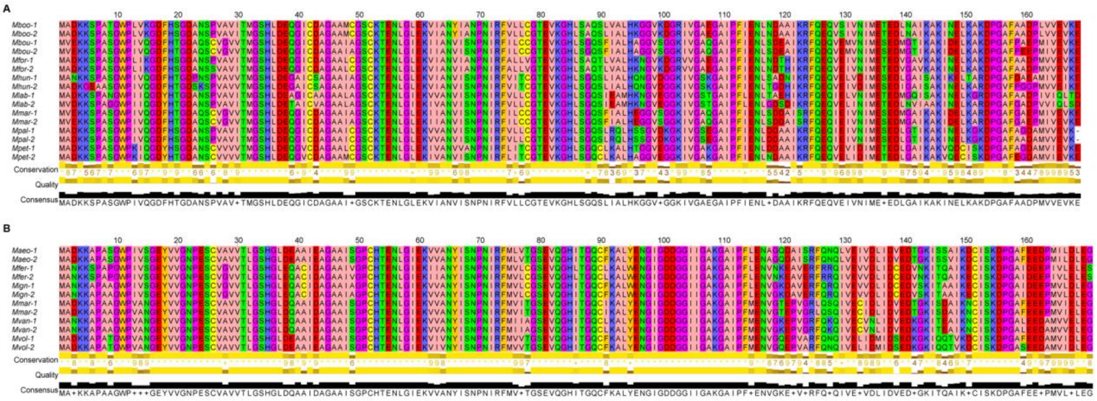

Figure S2 (Related to Figure 2). Amino acid sequence alignment of mtrA domainin (*A*) Methanomicrobiales and (*B*) Methanococcales.

Figure S3 (Related to Figure 2)

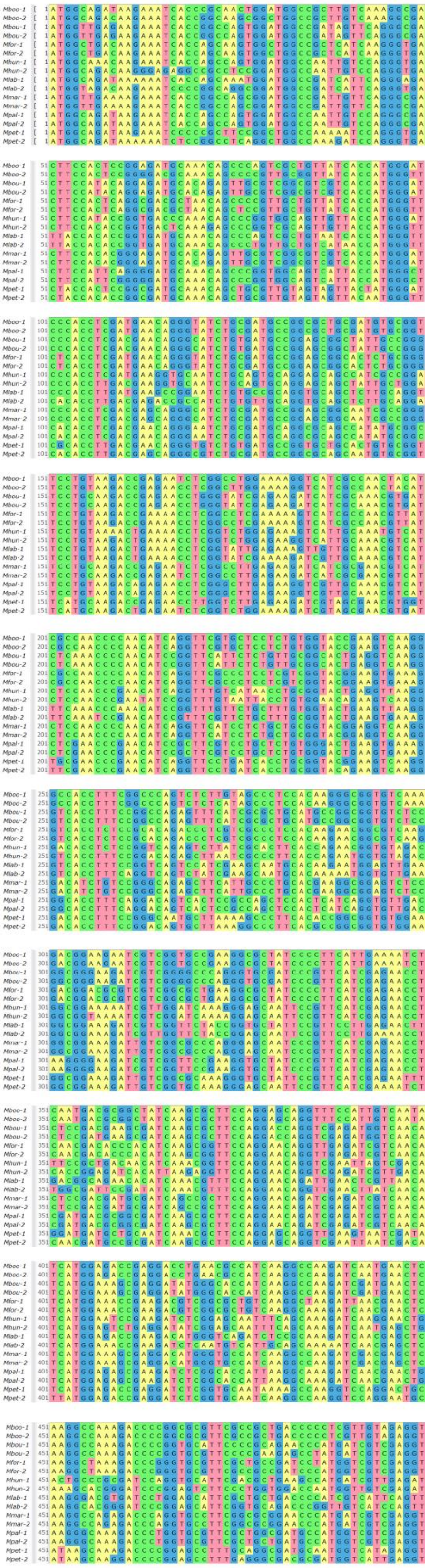

Figure S3 (Related to Figure 2): Nucleotide sequence alignment of mtrA domainin Methanomicrobiales.

Figure S4 (Related to Figure 2)

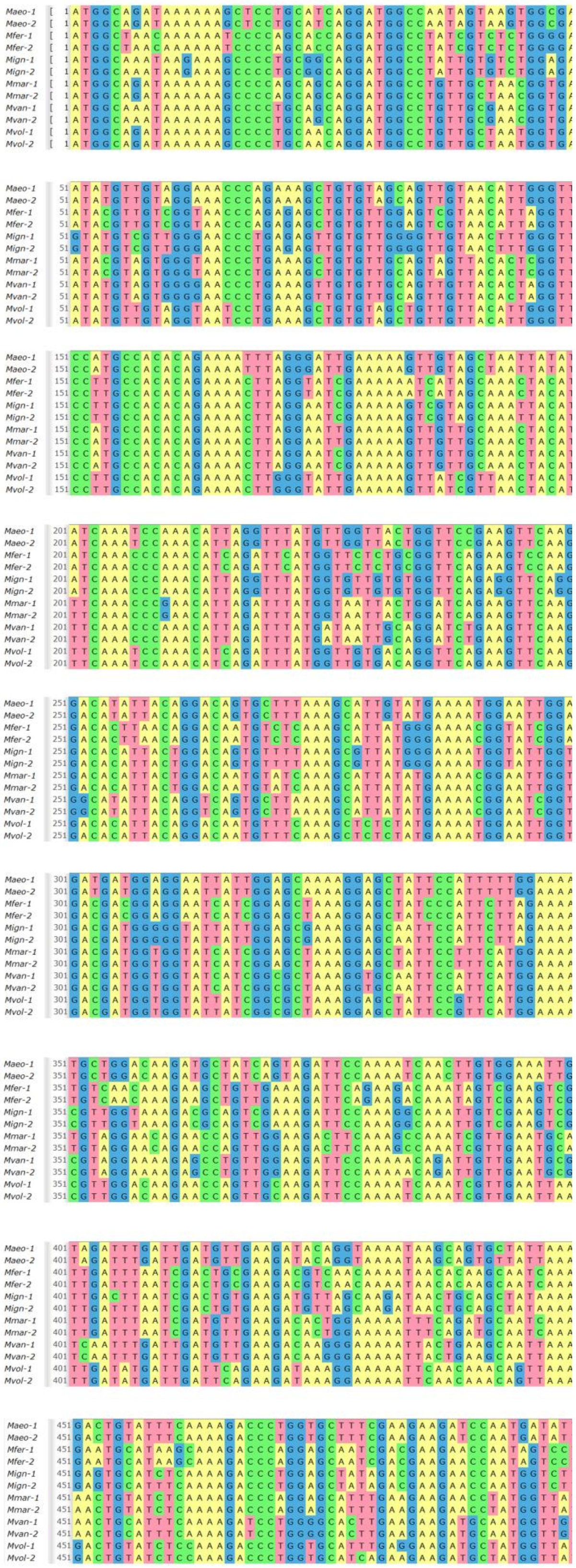

Figure S4 (Related to Figure 2): Nucleotide sequence alignment of mtrA domain in Methanococcales.

Figure S5 (Related to Figure 3)

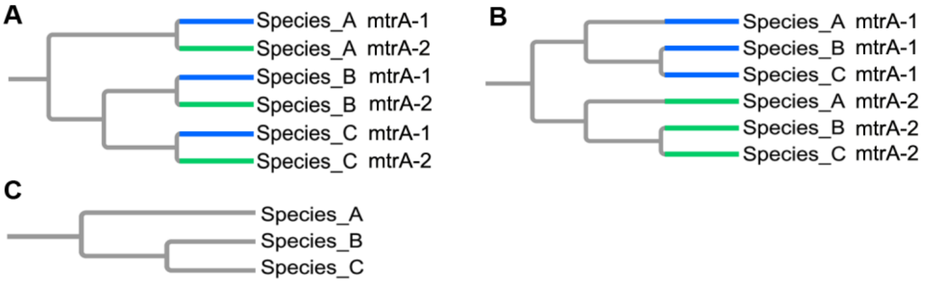

D

Methanomicrobiales										
	item	OBS*	AU	NP	BP	PP	KH	SH	WKH	WSH
	**topo A**	-1945.3	1	1	1	1	1	1	1	1
	**topo B**	1945.3	3.00E-40	2.00E-15	0	0	0	0	0	0
**Methanococcales**										
	item	OBS	AU	NP	BP	PP	KH	SH	WKH	WSH
	**topo A**	-2454.3	1	1	1	1	1	1	1	1
	**topo B**	2454.3	7.00E-77	8.00E-21	0	0	0	0	0	0

Figure S5 (Related to Figure 3 and Table S1). (*A*) The expected topology if gene conversion happens throughout all lineages. (*B*) The expected topology where no gene conversion happens. (*C*) The tree of species. (*D*) Statistic tests of two different topologies (*A* & *B*) of MtrA phylogeny in Methanomicrobiales and Methanococcales. *P*-values are calculated by different statistic tests in CONSEL for each topology. For simplicity, only three taxa are shown in each tree.

*Abbreviations of different tests:

OBS: Observation

AU: Approximately Unbiased test (Shimodaira 2002)

NP: Bootstrap Probability

BP: Non-scaled Bootstrap Probability

PP: Bayesian posterior probability

KH: Kishino-Hasegawa test (Kishino and Hasegawa 1989)

SH: Shimodaira-Hasegawa test (Shimodaira and Hasegawa 1999)

WKH: Weighted Kishino-Hasegawa test

WSH: Weighted Shimodaira-Hasegawa test

Figure S6 (Related to Figure 5)

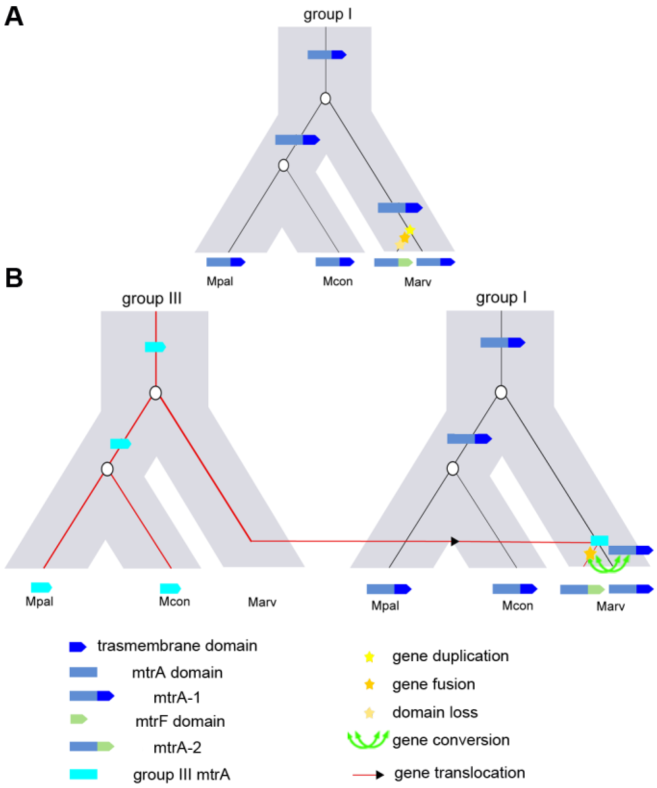

Figure S6 (Related to Figure 5). Two different scenarios in regards to the additional copy of group I *mtrA in Methanocella arvoryzae*. (*A*) The additional copy of group I *mtrA* (*mtrA-2*) in *Marv*s generated by a lineage-specific duplication of group I *mtrA* (yellow star), gene fusion (orange star) and the loss of the C-terminal transmembrane domain (light yellow star).(*B*) *mtrA-2* in *Marv*is generated via the translocation of group III *mtrA* followed by the fusion with *mtrF* and gene conversion (indicated as green arrow). Each branch in grey represents a speciation event. The abbreviation of species names are as follows: *Marv*, *Methanocella arvoryzae*; *Mcon*,*Methanocella conradii*; *Mpal*, *Methanocella paludicola*.

Figure S7 (Related to Figure 6)

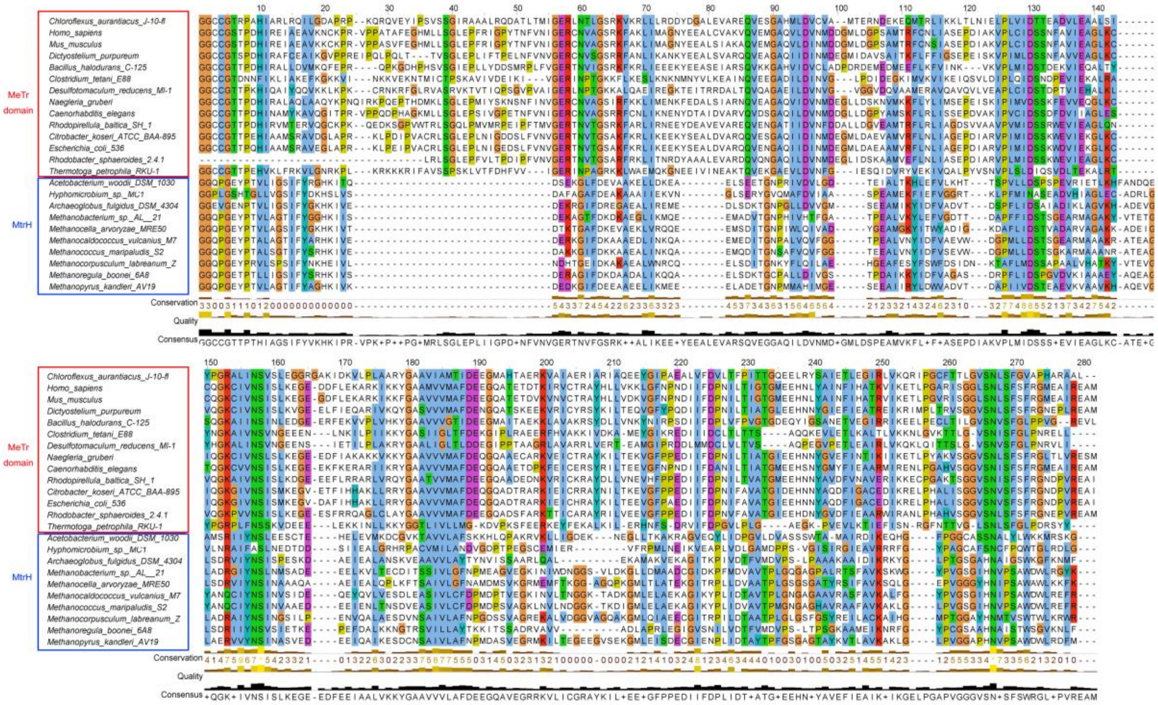

Figure S7 (Related to Figure 6). Multiple amino acid sequence alignment of MtrH and its remote homologs. Sequences whose names are in the blue box indicate MtrH. Those with names in the red box indicate MeTr domain.

Figure S8 (Related to Figure 6 & S7)

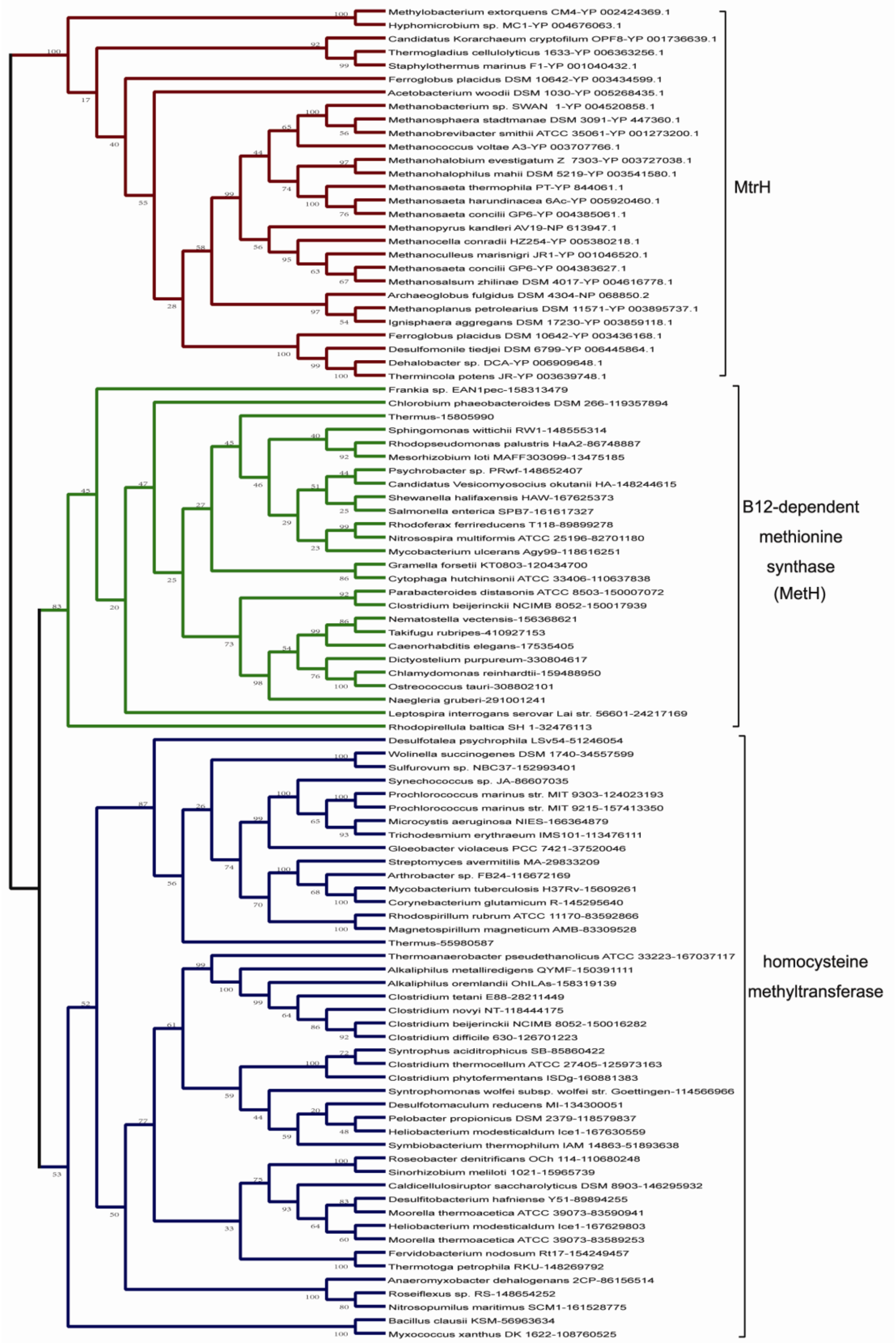

Figure S8 (Related to Figure 7). A ML tree of MtrH and MeTr domain-containing proteins. The numbers adjacent to the nodes denote the bootstrap values obtained from 500 times of bootstrap replicates.

Figure S9 (Related to Figure 4)

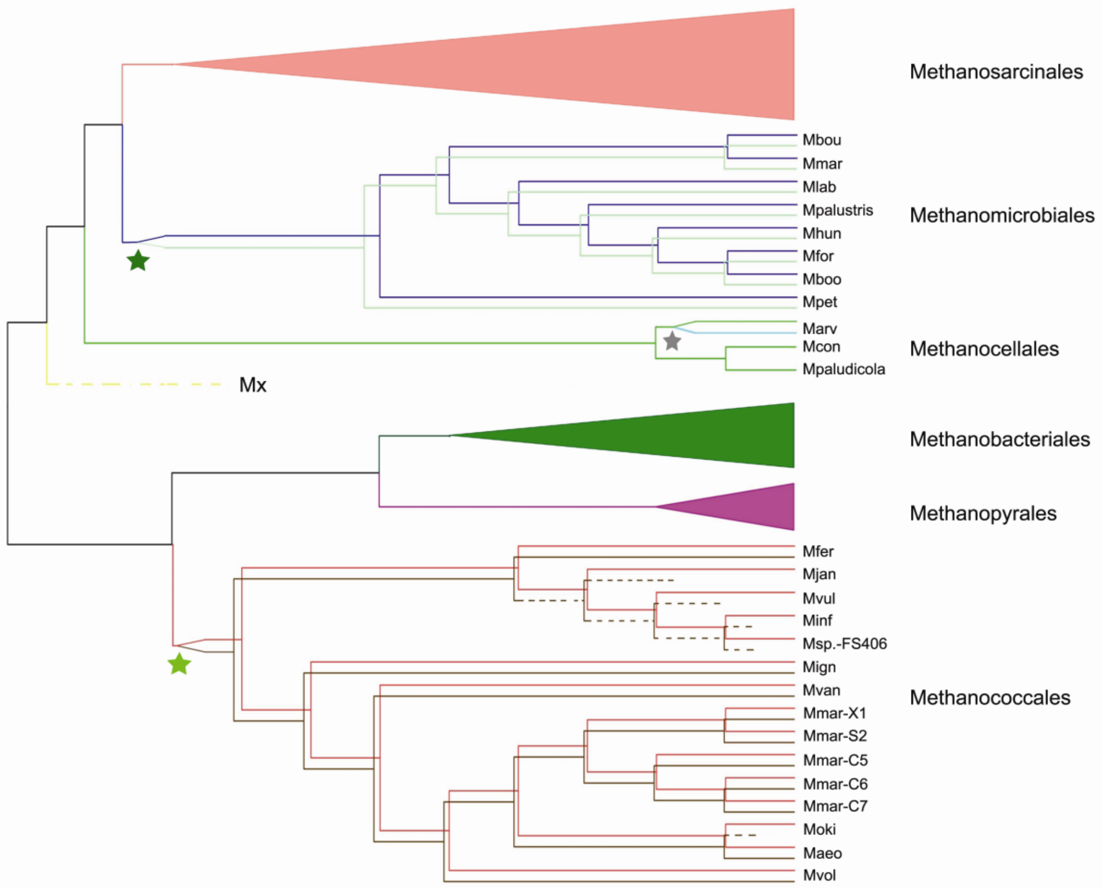

Figure S9 (Related to Figure 4). An overview of the evolutionary scenario of *mtrA-1/2* in all methanogens. Stars denote the formation of the fusion type of *mtrA* by duplication and domain shuffling in the ancestor of Methanomicrobiales, the ancestor of Methanococcales and species *Methanocella arvoryzae* independently. *mtrA-1/2* are indicated by two lines in parallel. Dashed lines indicate the loss of *mtrA*.

Supplementary References

Bagnall RD, Ayres KL, Green PM, Giannelli F. 2005. Gene conversion and evolution of Xq28 duplicons involved in recurring inversions causing severe hemophilia A. Genome Res 15:214-223.

Bergholz TM, Tarr CL, Christensen LM, Betting DJ, Whittam TS. 2007. Recent gene conversions between duplicated glutamate decarboxylase genes (gadA and gadB) in pathogenic Escherichia coli. Mol Biol Evol 24:2323-2333.

Carmon A, Wilkin M, Hassan J, Baron M, MacIntyre R. 2007. Concerted evolution within the drosophila dumpy gene. Genetics 176:309-325.

Chen S, Armistead JS, Provost-Javier KN, Sakamoto JM, Rasgon JL. 2010. Duplication, concerted evolution and purifying selection drive the evolution of mosquito vitellogenin genes. BMC Evol Biol 10.

Ezawa K, Ikeo K, Gojobori T, Saitou N. 2010. Evolutionary Pattern of Gene Homogenization between Primate-Specific Paralogs after Human and Macaque Speciation Using the 4-2-4 Method. Mol Biol Evol 27:2152-2171.

Ezawa K, OOta S, Saitou N. 2006. Genome-wide search of gene conversions in duplicated genes of mouse and rat. Mol Biol Evol 23:927-940.

Gao LZ, Innan H. 2004. Very low gene duplication rate in the yeast genome. Science 306:1367-1370.

Garb JE, DiMauro T, Lewis RV, Hayashi CY. 2007. Expansion and intragenic homogenization of spider silk genes since the triassic: Evidence from mygalomorphae (Tarantulas and their kin) spidroins. Mol Biol Evol 24:2454-2464.

Gerrard DT, Meyer A. 2007. Positive selection and gene conversion in SPP120, a fertilization-related gene, during the east African Cichlid fish radiation. Mol Biol Evol 24:2286-2297.

Kondrashov FA, Gurbich TA, Vlasov PK. 2007. Selection for functional uniformity of tuf duplicates in gamma-proteobacteria. Trends Genet 23:215-218.

Lathe WC, Bork P. 2001. Evolution of tuf genes: ancient duplication, differential loss and gene conversion. FEBS Lett 502:113-116.

Nystedt B, Frank AC, Thollesson M, Andersson SGE. 2008. Diversifying selection and

concerted evolution of a type IV secretion system in Bartonella. Mol Biol Evol 25:287-300.

Peel AD, Telford MJ, Akam M. 2006. The evolution of hexapod engrailed-family genes: evidence for conservation and concerted evolution. Proceedings Of the Royal Society B-Biological Sciences 273:1733-1742.

Privman E, Wurm Y, Keller L. 2013. Duplication and concerted evolution in a master sex determiner under balancing selection. Proceedings Of the Royal Society B-Biological Sciences 280.

Schmieder S, Colinet D, Poirie M. 2012. Tracing back the nascence of a new sex-determination pathway to the ancestor of bees and ants. Nat Commun 3:895.

Thomas JH. 2006. Concerted evolution of two novel protein families in Caenorhabditis species. Genetics 172:2269-2281.

Wang XX, Mitra N, Cruz P, Deng LW, Varki N, Angata T, Green ED, Mullikin J, Hayakawa T, Varki A, et al. 2012. Evolution of Siglec-11 and Siglec-16 Genes in Hominins. Mol Biol Evol 29:2073-2086.

Wang XY, Tang HB, Bowers JE, Paterson AH. 2009. Comparative inference of illegitimate recombination between rice and sorghum duplicated genes produced by polyploidization. Genome Res 19:1026-1032.
