# Supplementary material for: Long-Lasting Gene Conversion Shapes the Convergent Evolution of the Critical Methanogenesis Genes
Source: G3 (Bethesda). 2015 Sep 16;5(11):2475–86. doi: 10.1534/g3.115.020180 (PMC4632066; doi:10.1534/g3.115.020180)
Supplement: Supporting Information [file supp_g3.115.020180_TableS1.pdf]

Table S1 (Related to Figure 3&4): Gene conversion events detected by GENECONV between *mtrA-1* and *mtrA-2* paralogous genes in different species. *P*-value obtained from 100,000 permutations is shown as indicator of the confidence level of each gene conversion event. Only those with *P*-values lower than 0.05 are considered as potential gene conversion events and summarized in the table. * SIM (Similarity), BC KA (Bonferroni-corrected Karlin-Altschul)

Organisms	SIM* *P*-value	BC KA *P*-value	Aligned begin	Aligned end	Length	No. of polymorphisms	Total differences	Mismatch penalty
Methanoculleus marisnigri JR1	0	0	1	503	503	268	103	None
Methanosphaerula palustris E1_9c	0	0	61	478	418	222	116	None
Methanoregula formicicum SMSP	0	0	77	434	358	187	114	None
Methanoculleus bourgensis MS2	0	0	1	356	356	182	112	None
Methanoregula boonei 6A8	0	0	275	524	250	154	119	None
Methanocaldococcus fervens AG86	0	0	1	532	532	268	113	None
Methanococcus voltae A3	0	0	1	490	490	242	109	None
Methanococcus vannielii SB	0	0	1	551	551	287	93	None
Methanococcus maripaludis C5	0	0	1	551	551	287	89	None

Methanococcus maripaludis S2	0	0	1	551	551	287	88	None
Methanococcus maripaludis X1	0	0	1	534	534	270	89	None
Methanococcus aeolicus Nankai_3	0	0	1	454	454	219	105	None
Methanotorris igneus Kol 5	0	0	1	416	416	194	108	None
Methanococcus maripaludis C7	0.0031	0.0105 1	288	362	75	35	157	None
