# Supplementary material for: Long-Lasting Gene Conversion Shapes the Convergent Evolution of the Critical Methanogenesis Genes
Source: G3 (Bethesda). 2015 Sep 16;5(11):2475–86. doi: 10.1534/g3.115.020180 (PMC4632066; doi:10.1534/g3.115.020180)
Supplement: Supporting Information [file supp_g3.115.020180_TableS2.pdf]

Table S2 (Related to Figure 3&4): Recombination tests by different algorithms implemented in the RDP4 package. The *P*-value is shown if a recombination between two paralogs from the same species is detected for each algorithm. Otherwise indicated as 'NS' (not significant).

Species	RDP	Bootscan	Maxchi	Chimaera	SiSscan	3Seq
Methanoculleus marisnigri JR1	7.42E-23	7.74E-14	1.04E-18	7.24E-16	2.13E-19	8.76E-32
Methanosphaerula palustris E1-9C	3.98E-20	7.68E-24	2.80E-16	2.05E-16	1.55E-15	5.17E-31
Methanoregula formicicum SMSP	1.52E-18	1.01E-23	9.22E-19	2.39E-16	4.00E-19	4.34E-32
Methanoregula boonei 6A8	4.25E-15	5.40E-17	3.22E-17	7.90E-17	4.71E-11	7.17E-27
Methanoplanus petrolearius DSM	1.10E-09	3.84E-08	1.29E-09	1.71E-11	2.99E-08	6.22E-14
Methanocorpusculum labreanum Z	4.25E-08	2.17E-05	1.21E-12	5.06E-11	1.11E-06	3.10E-15
Methanospirillum hungatei JF-1	9.57E-06	0.000143	2.73E-08	3.56E-10	3.45E-10	8.46E-10
Methanocaldococcus fervens AG86	2.22E-22	4.26E-28	7.59E-19	1.51E-14	7.46E-21	2.88E-25
Methanotorris igneus Kol 5	5.27E-22	1.07E-18	5.72E-20	3.74E-14	3.94E-23	3.96E-26
Methanococcus maripaludis S2	3.89E-20	1.41E-16	2.83E-17	5.29E-17	1.29E-11	6.00E-29
Methanococcus vannielii SB	1.66E-17	3.43E-07	1.82E-12	1.61E-14	6.49E-18	2.72E-21
Methanococcus	2.99E-10	1.21E-12	5.86E-16	4.80E-13	9.45E-16	2.68E-15

voltae A3						
Methanococcus aeolicus Nankai-3	1.26E-09	4.06E-12	3.97E-05	3.01E-11	2.49E-25	2.47E-10
