# Supplementary material for: Long-Lasting Gene Conversion Shapes the Convergent Evolution of the Critical Methanogenesis Genes
Source: G3 (Bethesda). 2015 Sep 16;5(11):2475–86. doi: 10.1534/g3.115.020180 (PMC4632066; doi:10.1534/g3.115.020180)
Supplement: Supporting Information [file supp_g3.115.020180_TableS4.pdf]

Table S4 (Related to Figure 3 and 4): A summary of the duration of some known gene conversion events. The duration time of each gene conversion event was estimated based on TreeTime if colored in blue, or from the indicated literatures.

**Genes**	**Domain**	**Duration of gene conversion (Myr)**	**Literatures**
***mtrA-1/mtrA-2a* in Methanococcales**	**Archaea**	**> 2216**	**This study**
***mtrA-1/mtrA-2b* in Methanomicrobials**	**Archaea**	**> 2410**	**This study**
tuf	Bacteria	> 2500	(Lathe and Bork 2001; Kondrashov, Gurbich and Vlasov 2007)
gadA/B	Bacteria	< 100	(Bergholz, et al. 2007)
type IV secretion system genes	Bacteria	< 0.5	(Nystedt, et al. 2008)
engrailed-family genes in beetles	Eukarya	~ 360	(Peel, Telford and Akam 2006)
silk genes in spiders	Eukarya	> 240	(Garb, et al. 2007)
Csd in ant/bee	Eukarya	115	(Schmieder, Colinet and Poirie 2012; Privman, Wurm and Keller 2013)
spp120 in Cichlid fish	Eukarya	100	(Gerrard and Meyer 2007)
vitellogenin genes in mosquito	Eukarya	100	(Chen, et al. 2010)
Nspb and *Nspc* gene families in C. elegans	Eukarya	50	(Thomas 2006)
dumpy genes in Drosophila	Eukarya	30	(Carmon, et al. 2007)
xq28 in primates	Eukarya	25	(Bagnall, et al. 2005)

siglec-11/16 in Hominins	Eukarya	1-1.2	(Wang, et al. 2012)
