# Supplementary material for: Long-Lasting Gene Conversion Shapes the Convergent Evolution of the Critical Methanogenesis Genes
Source: G3 (Bethesda). 2015 Sep 16;5(11):2475–86. doi: 10.1534/g3.115.020180 (PMC4632066; doi:10.1534/g3.115.020180)
Supplement: Supporting Information [file supp_g3.115.020180_TableS5.pdf]

Table S5 (Related to Figure 3 and 4): A summary of gene conversion events in genome-wide assays. The duration time of gene conversion in each organism was estimated based on TreeTime if colored in blue, or from the indicated literatures.

**Taxa**	**No. of gene pairs/gene families**	**gene conversion lasting time (Myr)**	**Literatures**
yeast	55	< 100	(Gao and Innan 2004)
primates	43	< 20-95	(Ezawa, et al. 2010)
rice	244	< 41.3	(Wang, et al. 2009)
sorghum	210	< 41.3	(Wang, et al. 2009)
rat and mouse	488	< 25.4	(Ezawa, OOta and Saitou 2006)
