# Supplementary material for: Long-Lasting Gene Conversion Shapes the Convergent Evolution of the Critical Methanogenesis Genes
Source: G3 (Bethesda). 2015 Sep 16;5(11):2475–86. doi: 10.1534/g3.115.020180 (PMC4632066; doi:10.1534/g3.115.020180)
Supplement: Supporting Information [file supp_g3.115.020180_FigureS1.pdf]

Figure S1 (Related to Figure 1)

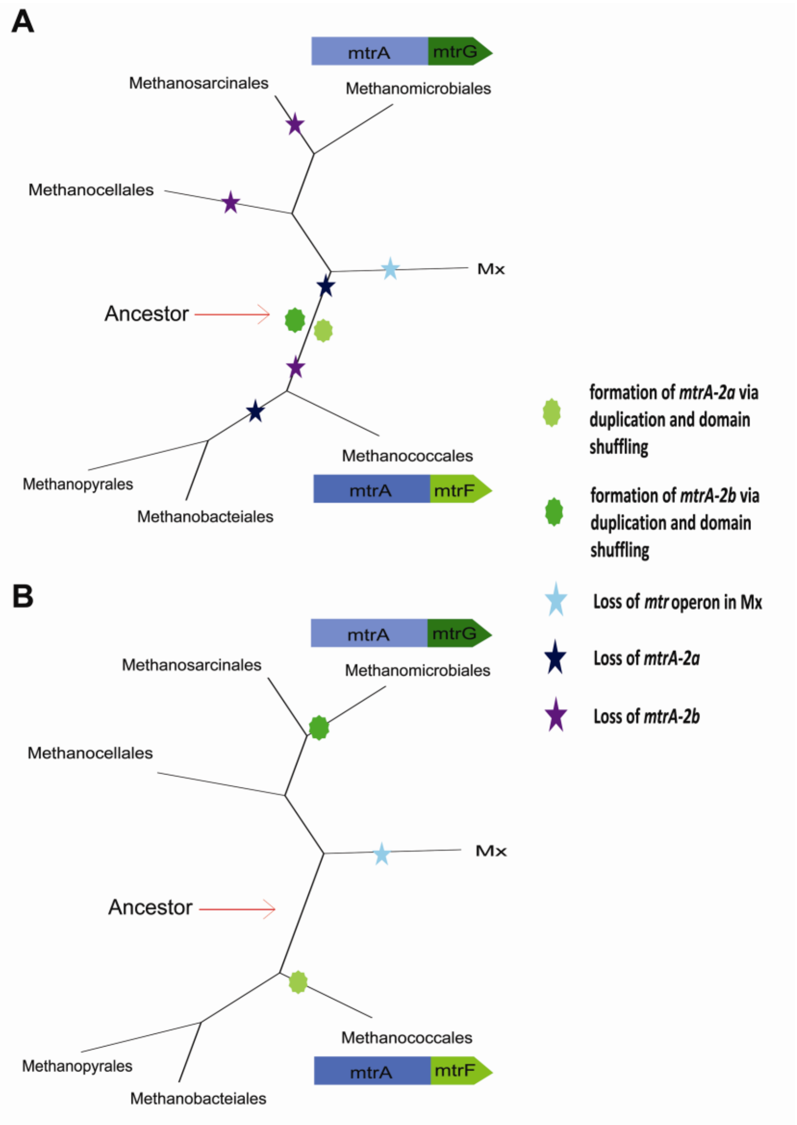

Figure S1 (Related to Figure 1). Evolutionary scenarios of the convergent evolutionary pattern of *mtrA-2*. (A) Scenario I: *mtrA-2* originated in Methanomicrobiales and Methanococcales independently. (B) Scenario II: *mtrA-2* originated in the common ancestor of all methanogens followed by several gene loss events in different lineages for at least five times.
