# Supplementary material for: Long-Lasting Gene Conversion Shapes the Convergent Evolution of the Critical Methanogenesis Genes
Source: G3 (Bethesda). 2015 Sep 16;5(11):2475–86. doi: 10.1534/g3.115.020180 (PMC4632066; doi:10.1534/g3.115.020180)
Supplement: Supporting Information [file supp_g3.115.020180_FigureS5.pdf]

Figure S5 (Related to Figure 3)

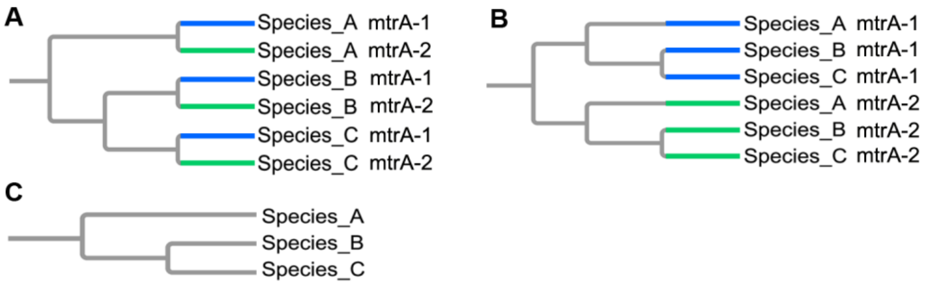

D

Methanomicrobiales										
	item	OBS*	AU	NP	BP	PP	KH	SH	WKH	WSH
	**topo A**	-1945.3	1	1	1	1	1	1	1	1
	**topo B**	1945.3	3.00E-40	2.00E-15	0	0	0	0	0	0
**Methanococcales**										
	item	OBS	AU	NP	BP	PP	KH	SH	WKH	WSH
	**topo A**	-2454.3	1	1	1	1	1	1	1	1
	**topo B**	2454.3	7.00E-77	8.00E-21	0	0	0	0	0	0

Figure S5 (Related to Figure 3 and Table S1). (*A*) The expected topology if gene conversion happens throughout all lineages. (*B*) The expected topology where no gene conversion happens. (*C*) The tree of species. (*D*) Statistic tests of two different topologies (*A* & *B*) of MtrA phylogeny in Methanomicrobiales and Methanococcales. *P*-values are calculated by different statistic tests in CONSEL for each topology. For simplicity, only three taxa are shown in each tree.

*Abbreviations of different tests:

OBS: Observation

AU: Approximately Unbiased test (Shimodaira 2002)

NP: Bootstrap Probability

BP: Non-scaled Bootstrap Probability

PP: Bayesian posterior probability

KH: Kishino-Hasegawa test (Kishino and Hasegawa 1989)

SH: Shimodaira-Hasegawa test (Shimodaira and Hasegawa 1999)

WKH: Weighted Kishino-Hasegawa test

WSH: Weighted Shimodaira-Hasegawa test
