# Supplementary material for: Long-Lasting Gene Conversion Shapes the Convergent Evolution of the Critical Methanogenesis Genes
Source: G3 (Bethesda). 2015 Sep 16;5(11):2475–86. doi: 10.1534/g3.115.020180 (PMC4632066; doi:10.1534/g3.115.020180)
Supplement: Supporting Information [file supp_g3.115.020180_FigureS6.pdf]

Figure S6 (Related to Figure 5)

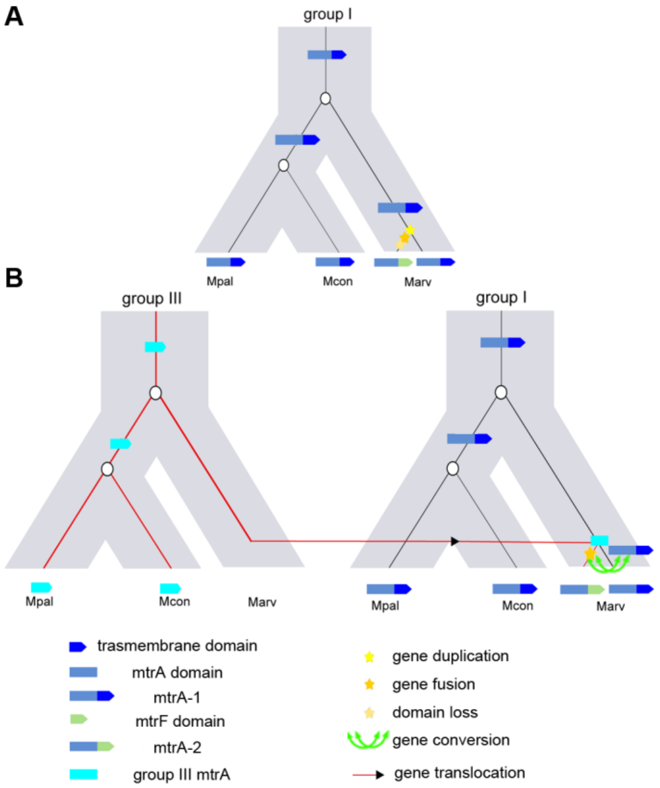

Figure S6 (Related to Figure 5). Two different scenarios in regards to the additional copy of group I *mtrA in Methanocella arvoryzae*. (*A*) The additional copy of group I *mtrA* (*mtrA-2*) in *Marv*s generated by a lineage-specific duplication of group I *mtrA* (yellow star), gene fusion (orange star) and the loss of the C-terminal transmembrane domain (light yellow star).(*B*) *mtrA-2* in *Marv*is generated via the translocation of group III *mtrA* followed by the fusion with *mtrF* and gene conversion (indicated as green arrow). Each branch in grey represents a speciation event. The abbreviation of species names are as follows: *Marv*, *Methanocella arvoryzae*; *Mcon*,*Methanocella conradii*; *Mpal*, *Methanocella paludicola*.
