# Supplementary material for: Long-Lasting Gene Conversion Shapes the Convergent Evolution of the Critical Methanogenesis Genes
Source: G3 (Bethesda). 2015 Sep 16;5(11):2475–86. doi: 10.1534/g3.115.020180 (PMC4632066; doi:10.1534/g3.115.020180)
Supplement: Supporting Information [file supp_g3.115.020180_FigureS7.pdf]

Figure S7 (Related to Figure 6)

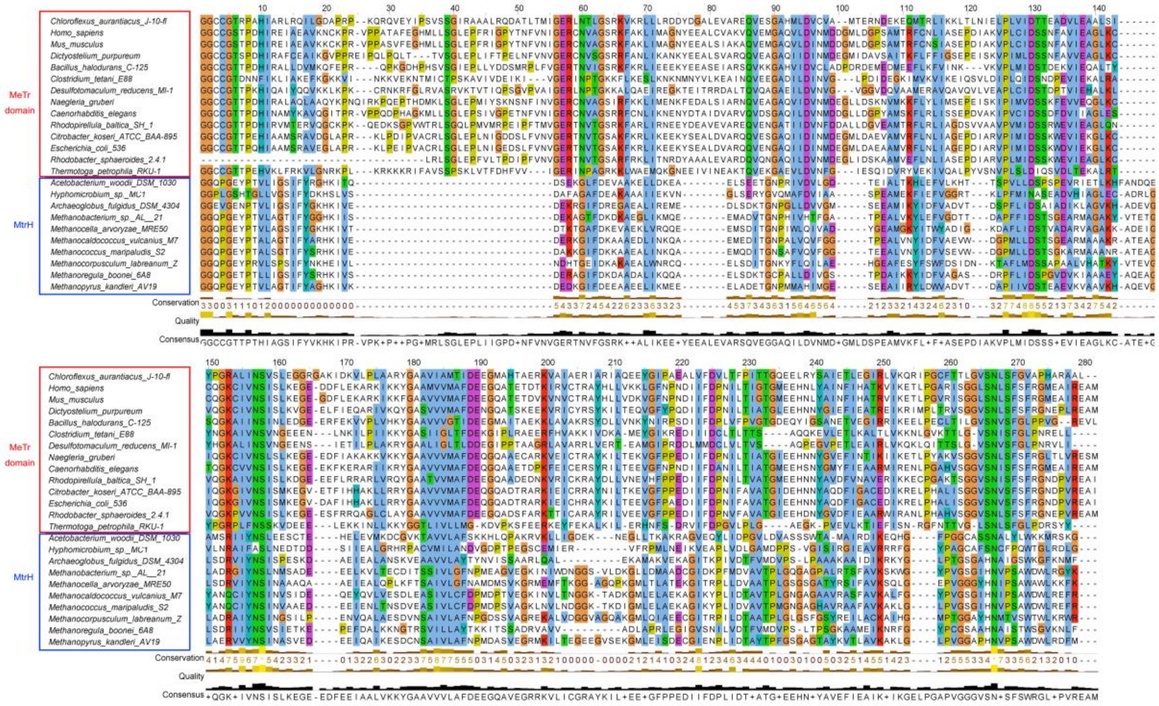

Figure S7 (Related to Figure 6). Multiple amino acid sequence alignment of MtrH and its remote homologs. Sequences whose names are in the blue box indicate MtrH. Those with names in the red box indicate MeTr domain.
