# Supplementary material for: Long-Lasting Gene Conversion Shapes the Convergent Evolution of the Critical Methanogenesis Genes
Source: G3 (Bethesda). 2015 Sep 16;5(11):2475–86. doi: 10.1534/g3.115.020180 (PMC4632066; doi:10.1534/g3.115.020180)
Supplement: Supporting Information [file supp_g3.115.020180_FigureS8.pdf]

Figure S8 (Related to Figure 6 & S7)

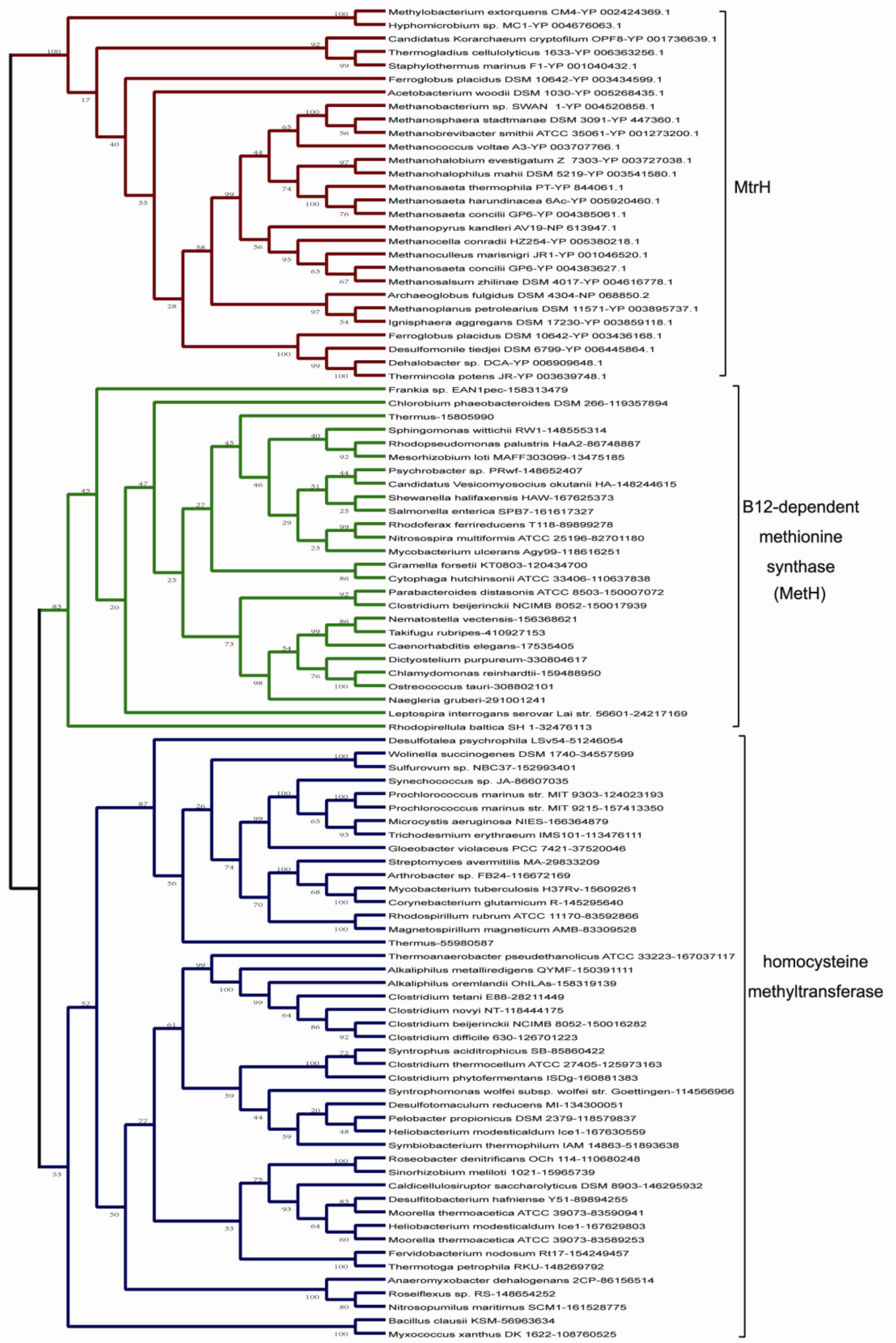

Figure S8 (Related to Figure 7). A ML tree of MtrH and MeTr domain-containing proteins. The numbers adjacent to the nodes denote the bootstrap values obtained from 500 times of bootstrap replicates.
