# Supplementary material for: Long-Lasting Gene Conversion Shapes the Convergent Evolution of the Critical Methanogenesis Genes
Source: G3 (Bethesda). 2015 Sep 16;5(11):2475–86. doi: 10.1534/g3.115.020180 (PMC4632066; doi:10.1534/g3.115.020180)
Supplement: Supporting Information [file supp_g3.115.020180_FigureS9.pdf]

Figure S9 (Related to Figure 4)

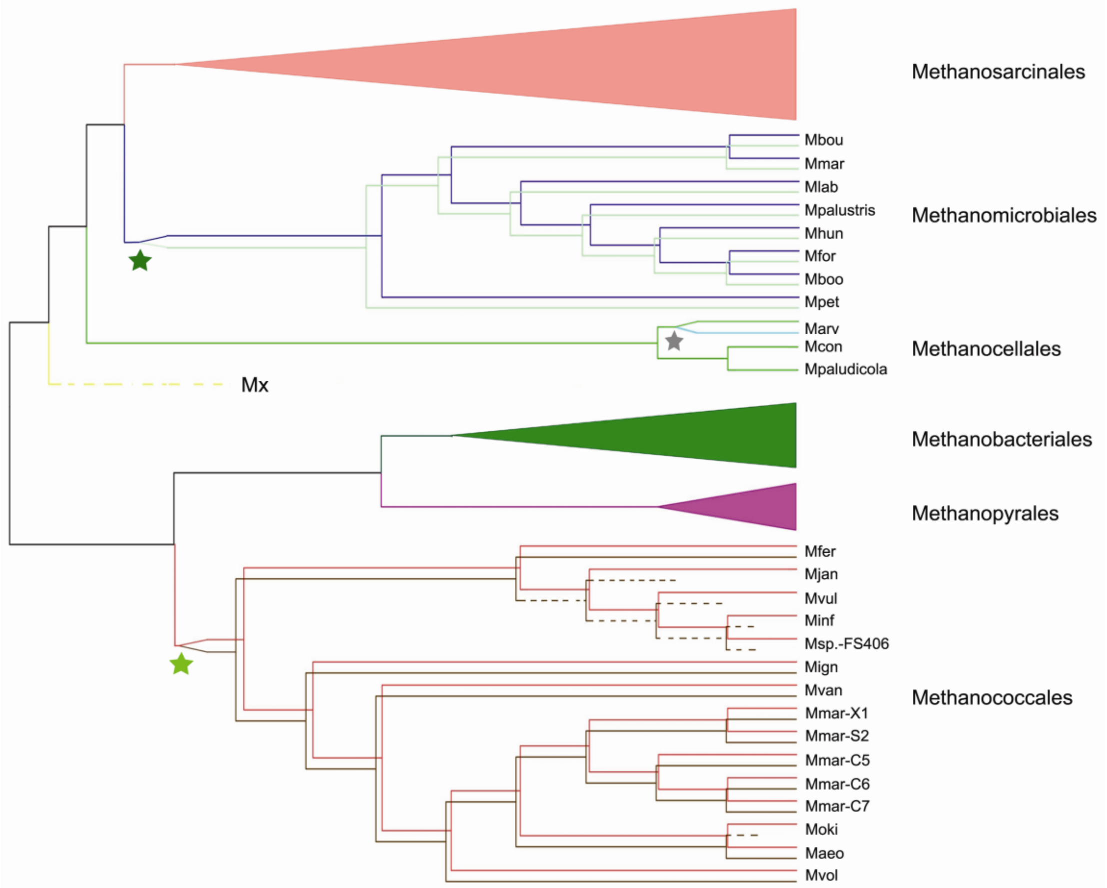

Figure S9 (Related to Figure 4). An overview of the evolutionary scenario of *mtrA-1/2* in all methanogens. Stars denote the formation of the fusion type of *mtrA* by duplication and domain shuffling in the ancestor of Methanomicrobiales, the ancestor of Methanococcales and species *Methanocella arvoryzae* independently. *mtrA-1/2* are indicated by two lines in parallel. Dashed lines indicate the loss of *mtrA*.
